# Longitudinal variation in human immunodeficiency virus long terminal repeat methylation in individuals on suppressive antiretroviral therapy

**DOI:** 10.1186/s13148-019-0735-9

**Published:** 2019-09-13

**Authors:** César N. Cortés-Rubio, Gonzalo Salgado-Montes de Oca, Francisco J. Prado-Galbarro, Margarita Matías-Florentino, Akio Murakami-Ogasawara, Leticia Kuri-Cervantes, Ana P. Carranco-Arenas, Christopher E. Ormsby, Ivette K. Cortés-Rubio, Gustavo Reyes-Terán, Santiago Ávila-Ríos

**Affiliations:** 1 Centre for Research in Infectious Diseases, National Institute of Respiratory Diseases, Tlalpan 4502, 14080 Mexico City, Mexico; 2 National Institute of Public Health, Mexico City, Mexico; 3 Department of Microbiology, Perelman School of Medicine, University of Pennsylvania, Philadelphia, PA USA; 4 Adolfo López Mateos Hospital, ISSSTE, Mexico City, Mexico

**Keywords:** HIV, 5′-LTR, Latency, DNA methylation, CpG, Longitudinal, Proviral load, Residual plasma viral load, Non-canonical methylation

## Abstract

**Background:**

Persistence of latent, replication-competent provirus in CD4^+^ T cells of human immunodeficiency virus (HIV)-infected individuals on antiretroviral treatment (ART) is the main obstacle for virus eradication. Methylation of the proviral 5′ long terminal repeat (LTR) promoter region has been proposed as a possible mechanism contributing to HIV latency; however, conflicting observations exist regarding its relevance. We assessed 5′-LTR methylation profiles in total CD4^+^ T cells from blood of 12 participants on short-term ART (30 months) followed up for 2 years, and a cross-sectional group of participants with long-term ART (6–15 years), using next generation sequencing. We then looked for associations between specific 5′-LTR methylation patterns and baseline and follow-up clinical characteristics.

**Results:**

5′-LTR methylation was observed in all participants and behaved dynamically. The number of 5′-LTR variants found per sample ranged from 1 to 13, with median sequencing depth of 16270× (IQR 4107×-46760×). An overall significant 5′-LTR methylation increase was observed at month 42 compared to month 30 (median CpG Methylation Index: 74.7% vs. 0%, *p* = 0.025). This methylation increase was evident in a subset of participants (methylation increase group), while the rest maintained fairly high and constant methylation (constant methylation group). Persons in the methylation increase group were younger, had higher CD4^+^ T cell gain, larger CD8% decrease, and larger CD4/CD8 ratio change after 48 months on ART (all *p* < 0.001). Using principal component analysis, the constant methylation and methylation increase groups showed low evidence of separation along time (factor 2: *p* = 0.04). Variance was largely explained (21%) by age, CD4^+^/CD8^+^ T cell change, and CD4^+^ T cell subpopulation proportions. Persons with long-term ART showed overall high methylation (median CpG Methylation Index: 78%; IQR 71–87%). No differences were observed in residual plasma viral load or proviral load comparing individuals on short-term (both at 30 or 42 months) and long-term ART.

**Conclusions:**

Our study shows evidence that HIV 5′-LTR methylation in total CD4+ T cells is dynamic along time and that it can follow different temporal patterns that are associated with a combination of baseline and follow-up clinical characteristics. These observations may account for differences observed between previous contrasting studies.

**Electronic supplementary material:**

The online version of this article (10.1186/s13148-019-0735-9) contains supplementary material, which is available to authorized users.

## Background

Modern combination antiretroviral treatment (ART) reduces human immunodeficiency virus (HIV) plasma viral load (pVL) to undetectable levels (< 40 HIV RNA copies/mL), achieving a 4–5 log_10_ reduction during the first year of treatment [1]. However, HIV proviral load is more stable with only 1 log_10_ reduction in the same treatment period [2]. Persistence of infectious provirus in CD4^+^ T cells (and maybe other cell lineages) in HIV-infected individuals on ART is the main obstacle for virus eradication [3]. The major HIV-1 reservoir is a small pool of latently infected resting memory CD4^+^ T cells [4], which are present in a frequency of 1/10^6^ resting CD4^+^ T cells in persons on suppressive ART [5]. "Kick and kill" HIV cure strategies aim to achieve complete activation of latently-infected cells so that they can be eliminated by diverse immunologic/pharmacologic strategies. To this day, these strategies have shown limited success [6].

Complicating HIV cure efforts, it is estimated that 88% of HIV proviruses are defective, while only 12% constitute intact proviruses [7], which in theory, would have the ability to activate and produce virions. Furthermore, ex vivo reactivation of only a small fraction of latent proviruses (0.12%) has been achieved from resting CD4^+^ T cells of individuals under ART using strategies based on vorinostat, a histone deacetylase inhibitor (HDACi) [6]. In vivo, treatment with panobinostat, another HDACi, did not achieve a cohort-wide reduction in total HIV DNA, integrated HIV DNA, proportion of cells carrying replication competent virus, or number of latently infected cells [8].

A plausible explanation of why certain proviruses fail to reactivate after stimulation may be due to the fact that HIV epigenetic silencing could be the product of a complex and heterogeneous pattern of histone marks [9–12], DNA methylation [13–19], the effect of chromatin remodeling complexes such as Polycomb [12, 20, 21] and BRG1-associated factors (BAF) [22, 23], and non-coding RNAs [24–28], not to mention genetic and microenvironment factors associated with the viral integration site [7]. Indeed, it has been observed that HDACi-based strategies to activate the latent HIV reservoir could paradoxically increase the degree of DNA methylation of the HIV 5′ long terminal repeat (LTR) and thus increase the stability of the reservoir [19]. Therefore, more profound knowledge on individual HIV latency mechanisms as well as on their interrelationships is warranted.

DNA methylation patterns are defined early in development and are closely related to both cell differentiation and gene expression; being modified in many disease states, including cancer, autoimmunity, and infectious diseases [29]. There is evidence that DNA from retroviruses integrated into mammalian genomes can become methylated de novo [30]. Furthermore, CpG methylation of retroviral promoters and enhancers located in the 5′-LTR has been correlated with silencing of several retroviruses such as human T cell leukemia virus type 1 [31], Moloney murine leukemia virus [32], Rous sarcoma virus [33], and human endogenous retroviruses [34]. These observations have inspired several research groups to explore the role of LTR methylation in HIV infection.

There are contrasting results regarding the role of methylation of the HIV 5′-LTR promoter region on provirus expression in individuals on suppressive ART. Early work suggested that resting memory CD4^+^ T cells of individuals on long-term ART (median 11.5 years) without detectable viremia frequently contained hypermethylated and activation-resistant 5′-LTR, as opposed to hypomethylated 5′-LTR in viremic individuals [13]. Nevertheless, another study challenged these observations, finding that 5′-LTR methylation is rare in resting CD4^+^ T cells of aviremic individuals on short-term ART (median 2.9 years) [17]. More recently, another study found low levels of 5′-LTR DNA methylation in resting CD4^+^ T cells of persons on ART for up to 3 years, but accumulation of 5′-LTR DNA methylation in the latent reservoir after long-term ART and suggested that the transient stimulation of cells harboring latent proviruses may contribute to 5′-LTR methylation [19]. More studies are warranted to determine to what extent 5′-LTR methylation plays a role on HIV latency in order to inform cure strategies.

In this work, we describe longitudinal changes in sequence, variant frequency, and methylation status of the HIV 5′-LTR region, observed in CD4^+^ T cells from blood samples of HIV-infected persons with a short history of suppressive ART compared to persons with long-term suppressive ART. We also studied possible associations between methylation patterns and baseline and follow-up clinical characteristics of the participants.

## Results

### Longitudinal 5′-LTR methylation profiles from total CD4^+^ T cells of persons with short-term ART

**Fig. 1:**
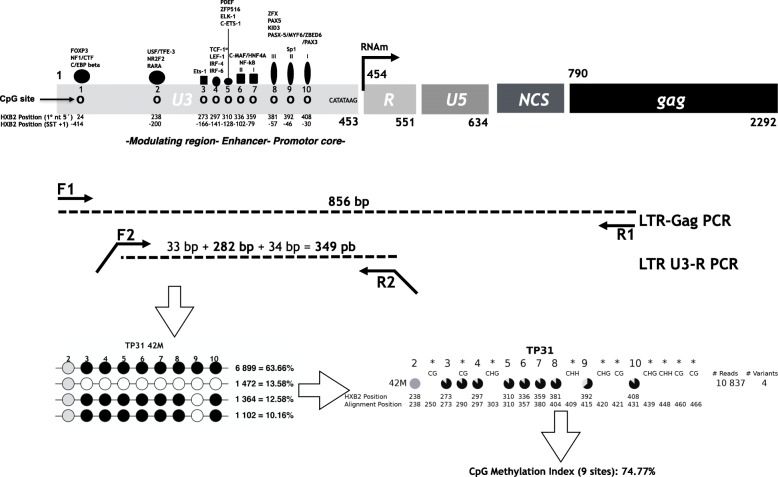
HIV 5′-LTR amplification and sequencing strategy. The 5′-LTR region (structure of the reference HXB2 sequence detailed in the figure, including canonical CpG and transcription factor binding sites) was amplified by nested PCR from total CD4^+^ T cell DNA treated with sodium bisulfite. The first-round PCR generated an 856-bp product including a fragment of *gag*, in order to selectively amplify the 5′-LTR and not the 3′-LTR. Nested PCR was performed with primers including Illumina adaptors for next generation sequencing. A 349-bp amplicon including nine of the canonical HXB2 CpG positions in the U3-R region was generated and deep sequenced. Assuming that each read corresponded to a single amplicon, we quantified, normalized, and aligned all variants obtained for each sample (the number of reads and proportion in the sample is indicated next to each variant). Each canonical CpG position is represented with a circle. Positions resistant to bisulfite transformation were assumed to be methylated (black) and positions susceptible to bisulfite transformation were assumed to be non-methylated (white). Positions with mutations causing loss of the CpG site were also identified (gray). We then generated a summary representation of all the variants observed in each sample, using a pie chart per each methylation-susceptible position (colors represent the proportion of methylated, non-methylated and mutated variants at that specific site in the sample as above), numbered according to order of appearance in HXB2. We also included additional CpG, CHG, and CHH methylation-susceptible sites, not observed in the HXB2 sequence (marked with *). Finally, we defined a CpG Methylation Index metric per sample, averaging the proportion of methylated variants in each of the nine 5′-LTR canonical CpG sites analyzed

**Fig. 2:**
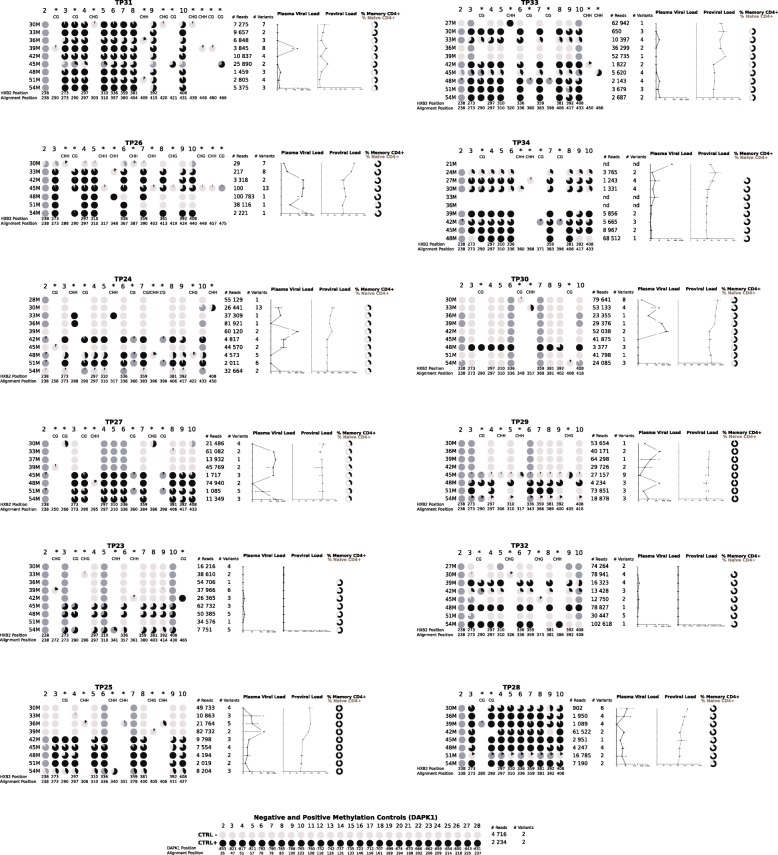
Overall longitudinal HIV 5′-LTR methylation profiles from total CD4^+^ T cells of 12 participants with short-term antiretroviral treatment. Each group represents a single participant on short-term antiretroviral treatment (24 to 30 months), followed up for 2 years at 3-month intervals. Each line depicts a summary of all variants observed per time point. CpG sites are shown as pie charts indicating the proportion of methylated (black), unmethylated (light gray) or mutated (dark gray) variants in the sample. Canonical HXB2 CpG positions are numbered. Additional CpG, CHG, and CHH methylation-susceptible sites, not observed in the HXB2 sequence, are also included (marked with *). The number of reads and the total number of variants obtained per time point, after filtering and aligning, are shown. Additionally, measurements of residual plasma viral load, proviral load and proportion of memory (CD3^+^/CD4^+^/CD28^+−^/CD95^+^; black) and naïve (CD3^+^/CD4^+^/CD45RO^−^/CCR7^+^/CD28+/CD95^−^; light gray) CD4^+^ T cells per time point are shown

**Fig. 3:**
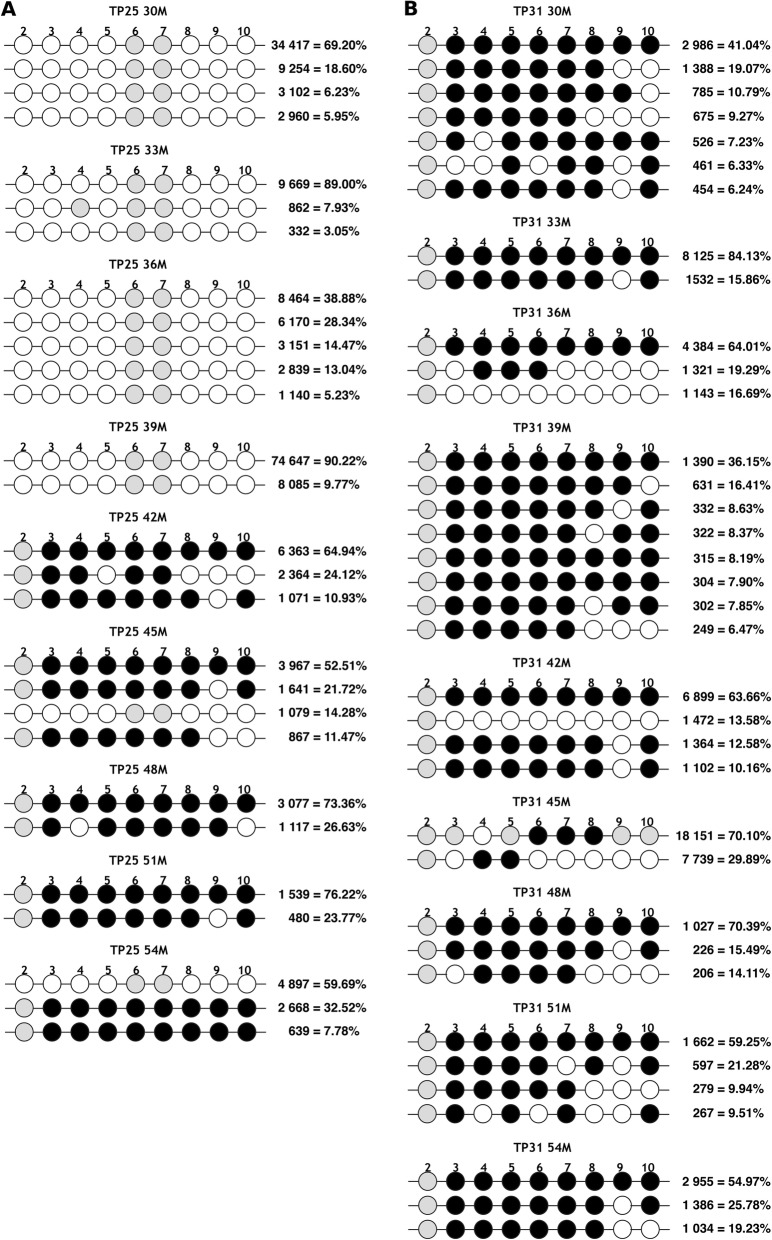
Detailed time trends of HIV 5′-LTR methylation in two model participants. Detailed variant composition per time point (30, 33, 36, 39, 42, 45, 48, 51, and 54 months) is shown for participants **a** TP25, representative of the methylation increase group, and **b** TP31, representative of the constant methylation group. Each horizontal line represents a variant. The number of reads representing that variant and its proportion in the sample are shown on the right. Each circle represents a canonical CpG site, numbered according to the HXB2 reference: black—methylated, white—non-methylated, gray—mutated

We assessed longitudinal methylation profiles based on nine canonical 5′-LTR CpG sites, present in the reference HXB2 sequence, in total CD4^+^ T cells from blood of 12 participants on short-term ART (24–30 months), using next generation sequencing, as explained in the “Methods”, (Fig. 1). For each participant, a 2-year follow up in 3-month intervals was performed (Fig. 2). To have a quantitative methylation measure, we estimated a “CpG Methylation Index,” averaging the weighed contribution of each CpG site to the overall 5′-LTR methylation of each sample. Additionally, for each time point, we determined proviral and residual plasma viral loads, and CD4^+^ T cell subpopulation distributions (defined as shown in Additional file 1: Figure S1). Participants were mostly male (92%), with a median age of 39 years (IQR 33–47) and a median CD4^+^ nadir of 71 cells/mm^3^ (IQR 7–251) (Table 1). Most persons on short-term ART (11/12) started with a tenofovir + emtricitabine + efavirenz regimen. Further, 7/12 participants remained with their original ART regimen throughout the follow-up period, 3/12 changed the backbone to abacavir + lamividune, and 3/12 changed the third drug from efavirenz to atazanarvir/ritonavir or nevirapine due to drug adverse effects (no drug resistance was observed in any of the participants) (Additional file 2: Table S1). The median number of reads obtained to assess viral variants was 16270 (IQR 4107–46760). The number of variants observed in each time point ranged from 1 to 13 and no association between the number of variants and the time on ART was observed in any of the participants (*p* > 0.05 in all cases). In general, methylation of the 5′-LTR was common and observed in at least one time point in all participants (Fig. 2). Moreover, 5′-LTR methylation showed a dynamic behavior over time. Overall, two general methylation patterns were observed: methylation remained present throughout the follow-up period (constant methylation group; e.g., participant TP31) or methylation significantly increased at some point (mainly around month 42) along the study period (methylation increase group; e.g., participant TP25) (Fig. 3). Examining individual variants, differences in the degree of methylation between different time points frequently coincided with alternation in predominance of heavily methylated vs. unmethylated variants (Fig. 3 and Additional file 3: Figure S2). In general, variants lacking methylated sites showed loss of one or more CpG sites by mutation, compared to HXB2 (e.g., Fig. 3, TP25 36M). Variants showing eight methylated canonical CpG sites (the CpG2 site was usually mutated or unmethylated) were common, observed in all participants in at least one time point.

**Table 1:** Baseline characteristics of the short-term constant methylation and methylation increase groups compared to the long-term ART group

	Short-Term ART group Combined *n* = 12	*p* value^a^	Short-Term ART group Constant methylation *n* = 5	*p* value^b^	Short-Term ART group Methylation increase *n* = 7	*p* value^c^	*p* value^d^	Long-term ART group *n* = 10
Sex (Male/Female)	11:1		5:0		6:1			9:1
Age (years)	39 (33–47)	*0.013*	41 (38–45)	0.910	34 (31–48)	*0.004*	< *0.001*	45 (39–56)
Nadir CD4^+^ T cell count (cells/mm^3^)	71 (7–251)	0.294	128 (12–264)	0.843	13 (4–212)	0.106	0.075	96 (47–234)
Pre-ART pVL (RNA copies/mL)	194,077 (67,293–285,046)	0.077	144,952 (90,736–243,202)	0.174	248219 (59,479–285,552)	*0.048*	0.205	47,800 (7581–154,809)
Delta CD4^+^ T cell count at 48 months (cells/mm^3^)	388 (174–502)	< *0.001*	330 (130–412)	0.843	455 (214–580)	*0.029*	< *0.001*	291 (193–341)
Delta CD4 % at 48 months	13 (11–20)	0.163	12 (10–15)	0.190	13 (12–32)	0.023	*0.041*	11 (6–14.5)
Delta CD8^+^ T cell count at 48 months (cells/mm^3^)	172 (− 104, 315)	0.053	102 (− 109, 246)	0.751	202 (− 89, 325)	0.105	*0.023*	− 85 (− 405, 331)
Delta CD8 % at 48 months	− 15 (− 24, − 5)	0.268	− 13 (− 17, 3)	*0.015*	− 24 (− 25, − 10)	0.162	< *0.001*	− 32 (− 46, − 9)
Delta CD4/CD8 at 48 months	0.40 (0.24–0.80)	0.058	0.33 (0.23–0.35)	0.668	0.53 (0.28–0.84)	*0.036*	< *0.001*	0.35 (0.25–0.55)
Days to undetectable pVL	183 (101–368)	0.287	186 (99–384)	0.551	180 (107–344)	0.771	0.205	148 (95–379)
Third drug in current ART regimen (NNRTIs / PIs)	83% / 17%	0.624	80% / 20%	1	86% / 14%	0.603	1	70% / 30%
Number of ART regimen changes	0 (0–1)	*0.027*	0 (0–0)	*0.003*	1 (0–1)	0.102	*0.008*	1 (0.8–2)

For each variable, median (interquartile range) is shown

*ART* antiretroviral therapy, *pVL* plasma viral load, *NNRTI* non-nucleoside reverse transcriptase inhibitors, *PI* protease inhibitors. Significant *p* values (*p* < 0.05) are shown in italic

^a^Short-term ART group vs. long-term ART group

^b^Constant methylation group vs. long-term ART group

^c^Methylation increase group vs. long-term ART group

^d^Constant methylation group vs. methylation increase group

Overall, the number of CpG sites in the studied 5′-LTR fragment ranged from 1 to 10 across the study participants and different time points. Over time, we observed mutations leading to both loss and gain of canonical CpG sites in predominant variants. Additionally, all participants gained potentially methylation-susceptible CpG, CHG, or CHH sites, originally absent in the reference HXB2 in at least one time point (Fig. 2).

Taken together, our results show that HIV 5′-LTR methylation is variable along time in total CD4^+^ T cells from blood of individuals under short-term ART and that methylation can be observed from early stages of ART initiation. Furthermore, at least two different temporal methylation variation patterns were observed among the participants.

### Temporal patterns of 5′-LTR methylation

**Fig. 4:**
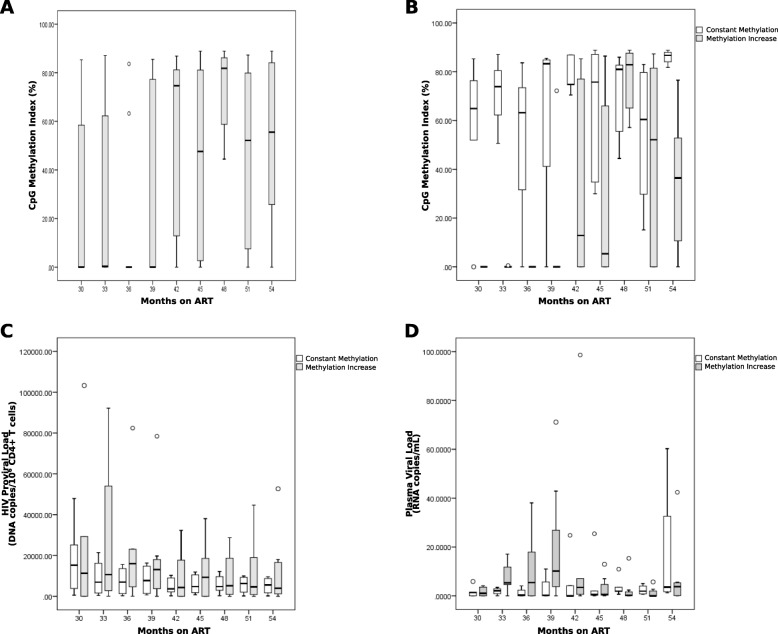
Changes in CpG Methylation Index, proviral load and residual plasma viral load along study follow-up. **a** The CpG Methylation Index was calculated as the average proportion of methylated variants considering the nine canonical HXB2 CpG sites analyzed, for each person and time point; all participants are included. **b** Participants were divided according to methylation patterns along time into two groups: constant methylation and methylation increase. CpG Methylation Index variations are shown for each group. **c** Proviral load variation along time for the constant methylation and methylation increase groups is shown. **d** Residual plasma viral load along time for the constant methylation and methylation increase groups is shown. In all cases, boxes represent 25–75% quartiles, whiskers 10–90% deciles, and horizontal lines within boxes represent medians

Considering all participants with short-term ART, we observed significant 5′-LTR methylation increase at month 42 with respect to month 30 (median CpG Methylation Index: 74.7% vs. 0%, *p* = 0.025) (Table 2, Fig. 4a). This methylation increase was evident in a subset of the participants (methylation increase group), while the rest maintained fairly high and constant methylation along the follow-up period (constant methylation group) (Fig. 2, 3, and 4b). Indeed, the methylation increase group was significantly different from the constant methylation group in that the starting 5′-LTR CpG methylation level (at 30 months on ART) in the former group was significantly lower compared to the latter (median CpG Methylation Index: 0 vs. 65, *p* = 0.007). This difference was also observed for methylation in non-canonical, additional CpG sites (median Additional CpG Methylation Index: 0 vs. 21.7 *p* = 0.030) (Table 3). Interestingly, we observed an overall decrease in proviral load at month 42 compared to month 30 (median proviral load: 3638 vs. 14146 copies/million CD4^+^ T cells, *p* = 0.005) (Table 2, Fig. 4c). Residual pVL was stable in most participants during follow-up, with occasional blips: TP30 39M (43 copies/mL), TP30 42M (99 copies/mL), TP24 39M (71 copies/mL), TP27 54M (42 copies/mL), and TP26 54M (60 copies/mL) (Fig. 2). No significant trends in time were observed for residual pVL. However, the constant methylation group showed overall less dispersion than the methylation increase group (except at month 54 when participant TP26 had a blip) (Fig. 4d).

**Table 2:** Early changes in methylation patterns and associated clinical variables

	Short-Term ART group (30 months)	*p* value^a^	Short-term ART group (42 months)	*p* value^b^	*p* value^c^	Long-term ART group
CpG methylation Index	0 (0–58.4)	*0.005*	74.7 (0–85.3)	0.273	*0.025*	77.9 (70.8–86.7)
Additional CpG methylation Index^d^	0.8 (0–21.0)	0.155	27.0 (4.3–45.1)	0.163	0.074	20.0 (13.8–20.0)
Non–CpG methylation Index^e^	1.3 (0–9.5)	0.388	0 (0–0)	0.332	0.093	0 (0–2.2)
Residual pVL (RNA copies/mL)	1.4 (0–3.6)	0.737	3.4 (0–7.1)	0.305	0.176	0.4 (0–2.9)
Proviral load (DNA copies/million CD4^+^ T cells)	14146 (517–29277)	0.526	3638 (228–10216)	0.260	*0.005*	9780 (4 057–13716)
Naive CD4^+^ T cells (%)	25.2 (20.6–52.6)	0.944	29.1 (18.3–49.1)	0.888	0.114	39.4 (11.2–45.4)
Memory CD4^+^ T cells (%)	74.8 (47.4–79.4)	0.944	70.9 (50.9–81.7)	0.888	0.114	60.6 (54.6–88.8)
T_SCM_ (%)	5.6 (3.0–6.9)	*0.049*	4.2 (2.9–7.1)	*0.035*	0.508	9.4 (5.7–15.9)
T_CM_ (%)	23.2 (15.3–31.2)	0.673	21.7 (12.7–33.0)	0.439	0.169	23.8 (19.5–40.9)
T_EM_ (%)	12.5 (2.1–20.4)	0.398	10.1 (3.2–12.5)	0.205	0.333	6.3 (4.8–9.3)
T_TM_ (%)	15.0 (11.3–25.4)	0.091	13.8 (11.4–31.5)	*0.049*	0.799	8.3 (4.5–16.6)
T_NEW_ (%)	1.1 (0.6–1.5)	*0.003*	2.5 (1.6–3.6)	0.526	*0.005*	2.0 (1.5–3.9)
T_TE_ (%)	2.3 (0.3–11.1)	0.181	5.0 (1.2–9.8)	0.944	0.285	4.8 (2.7–7.8)

In all cases, medians (interquartile ranges) are shown. Significant *p* values (*p* < 0.05) are shown in italic

*ART* antiretroviral therapy, *pVL* plasma viral load, *T*_*SCM*_ stem cell memory T cells, *T*_*CM*_ central memory T cells, *T*_*EM*_ effector memory T cells, *T*_*TM*_ transitional memory T cells, *T*_*NEW*_ T new, *T*_*TE*_ terminally differentiated effector T cells

^a^Short-term ART group (30 months) vs. long-term ART group

^b^Short-term ART group (42 months) vs. long-term ART group

^c^Short-term ART group (30 months) vs. short-term ART group (42 months)

^d^Considers methylation of CpG positions not present in the reference HXB2

^e^Considers methylation of non-CpG positions only

**Table 3:** Differential variation of clinical variables along time between the constant methylation and the methylation increase groups

	Short-term ART group (30 months)				Short-term ART group (42 months)				Long-term ART group
	Constant methylation group	*p* value^a^	Methylation increase group	*p* value^b^	Constant methylation group	*p* value^a^	Methylation increase group	*p* value^b^	
CpG methylation Index	64.9 (52.0–76.3)	0.244	0 (0–0)	*0.001*	74.8 (74.7–86.9)	0.902	12.9 (0–77.0)	0.071	77.9 (70.8–86.7)
Additional CpG methylation Index ^c^	21.7 (9.4–34.9)	0.282	0 (0–1.7)	*0.003*	27 (8.6–40.6)	0.282	27.0 (0–50.0)	0.219	20.0 (13.8–20.0)
Non–CpG methylation Index^d^	2.6 (0–8.3)	0.352	0 (0–11.2)	0.585	0 (0–3)	0.560	0 (0–0)	0.356	0 (0–2.2)
Residual pVL (RNA copies/ml)	1.4 (0–1.5)	0.699	1.0 (0–3.6)	0.862	0 (0–4.2)	0.842	3.5 (0.6–7.1)	0.164	0.4 (0–2.9)
Proviral load (DNA copies/million CD4^+^ T cells)	15241 (3800–25166)	0.462	11250 (22–29277)	0.745	3638 (2155–9012)	0.221	4264 (10–17670)	0.515	9780 (4057–13716)
Naive CD4^+^ T cells (%)	25.2 (23.9–45.7)	0.713	28.0 (4.2–64.1)	0.828	29.1 (27.7–49.1)	0.713	26.3 (4.8–47.0)	0.588	39.4 (11.2–45.4)
Memory CD4^+^ T cells (%)	74.8 (54.3-76.2)	0.713	72.1 (35.9–95.8)	0.828	70.9 (50.9–72.3)	0.713	73.7 (53.0–95.2)	0.588	60.6 (54.6–88.8)
T_SCM_ (%)	6.4 (4.7–6.6)	0.221	4.5 (1.0–6.9)	0.051	4.2 (2.9–7.4)	0.111	4.7 (3.4–6.2)	0.065	9.4 (5.7–15.9)
T_CM_ (%)	23.2 (23.2–28.8)	0.713	22.3 (15.3–35.1)	0.745	21.7 (12.7–33.0)	0.540	21.9 (15.5–32.5)	0.515	23.8 (19.5–40.9)
T_EM_ (%)	12.5 (5.4–17.2)	0.270	9.2 (2.1–26.5)	0.745	9.0 (7.6–10.1)	0.462	12.1 (3.2–13.8)	0.193	6.3 (4.8–9.3)
T_TM_ (%)	13.3 (12.6–15.1)	0.221	15.2 (11.3–25.4)	0.129	11.6 (11.4–19.5)	0.142	17.4 (11.7–31.5)	0.083	8.3 (4.5–16.6)
T_NEW_ (%)	1.4 (0.6–1.56)	0.075	1.1 (0.6–1.2)	*0.002*	2.5 (1.6–4.1)	0.624	2.7 (2.3–3.3)	0.588	2.0 (1.5–3.9)
T_TE_ (%)	4.0 (0.3–11.8)	0.713	1.5 (0.3–4.5)	0.083	3.9 (1.2–9.6)	0.806	6.3 (2.2–9.8)	0.914	4.8 (2.7–7.8)

In all cases, medians (interquartile ranges) are shown. Significant *p* values (*p* < 0.05) are shown in italic

*ART* antiretroviral therapy, *pVL* plasma viral load, *T*_*SCM*_ stem cell memory T cells, *T*_*CM*_ central memory T cells, *T*_*EM*_ effector memory T cells, *T*_*TM*_ transitional memory T cells, *T*_*NEW*_ T new, *T*_*TE*_ terminally differentiated effector T cells

^a^Constant methylation group vs. long-term ART group

^b^Methylation increase group vs. long-term ART group

^c^Considers methylation of CpG positions not present in the reference HXB2

^d^Considers methylation of non-CpG positions only.

A group of participants with a long history on ART was included to explore methylation associations with residual pVL and proviral load in the long term. This cross-sectional group included persons with median time on ART of 12 years (IQR 7–15), mostly male (90%), with median age of 45 years (IQR 39–56), and median nadir CD4^+^ T cell count of 96 cells/mm^3^ (IQR 47–234) (Table 1). Participants on long-term ART showed significantly more ART regimen changes than persons on short-term ART (8/10 participants changed ART regimen at least once), mostly for optimization or switches to newer drugs. The current ART regimen of participants in this group was tenofovir + emtricitabine + efavirenz for 7/10 and tenofovir + emtricitabine and a protease inhibitor for 3/10 (Additional file 2: Table S1). Persons with a long history of suppressive ART showed overall high degree of methylation with a median CpG Methylation Index of 78% (IQR 71–87%) (Table 2). Variants with mutated CpG sites were rare in this group and presence of new methylation-susceptible sites was infrequent, compared to individuals followed up longitudinally (Additional file 4: Figure S3).

Comparing the longitudinal follow-up group of participants at 30 months on ART with the cross-sectional group on long-term ART, we observed a significant difference in overall methylation (median CpG Methylation Index: 0% vs. 78%, *p* = 0.005), but this difference was lost at 42 months on ART (median CpG Methylation Index: 75% vs. 78%, *p* = 0.273) (Table 2). More specifically, the difference in early methylation (30 months vs. long-term ART) was observed in persons in the methylation increase group (*p* = 0.001), but not in the constant methylation group (*p* = 0.244) (Table 3). No differences were observed in residual pVL or proviral load, comparing individuals on short-term ART (both at 30 or 42 months of follow-up) and long-term ART (Tables 2 and 3).

In order to be more comprehensive in the methylation analysis, we studied non-canonical methylation patterns along the follow-up period, establishing a measure of methylation on CpG sites different to the canonical ones, present in the HXB2 reference (additional CpG methylation index), and methylation in sites other than CpG (non-CpG methylation index). Similar to our observations for canonical CpG methylation, a significant difference in additional CpG methylation was observed in persons in the methylation increase group (*p* = 0.003), but not in the constant methylation group (30 months; *p* = 0.282) when compared to persons on long-term ART (Table 3). No differences were observed in non-CpG methylation comparing the short-term (both at 30 and 42 months of follow-up) and long-term ART groups (Tables 2 and 3).

Considering CD4^+^ T cell subpopulation proportions, three general patterns were seen among participants: (1) individuals with higher proportion of naive CD4^+^ T cells (17%): TP24 and TP27; (2) individuals with higher proportion of memory CD4^+^ T cells (58%): TP26, TP23, TP25, TP34, TP29, TP32, and TP28; and (3) individuals with equal proportion of naive and memory CD4^+^ T cells (25%): TP31, TP33, and TP30; however, these groups did not show specific associations with methylation patterns (Fig. 2). Although no significant differences in the proportion of naïve and overall memory CD4^+^ T cells were observed between individuals with short-term and long-term ART, considering memory T cell subpopulations, a lower prevalence of the T_SCM_ and T_new_ was observed in individuals with 30 months on ART belonging to the methylation increase group (*p* = 0.051 and 0.002, respectively) compared to the long-term ART group, but not in the constant methylation group (*p* = 0.221 and 0.075, respectively) (Table 3), suggesting early differences in CD4^+^ T cell subpopulation composition between persons in the constant methylation and the methylation increase groups.

### Baseline and follow-up characteristics of persons with distinct 5′-LTR methylation patterns

Next, we looked for differences in the baseline characteristics of persons belonging to the constant methylation and the methylation increase groups (Table 1). Persons in the methylation increase group were significantly younger (median age: 34 vs. 41 years, *p* < 0.001), had higher CD4^+^ count gain at 48 months after ART initiation (delta CD4^+^ T cell count: 455 vs. 330, *p* < 0.001; delta CD4 %: 13 vs. 12, *p* = 0.041), higher CD8^+^ count change at 48 months (delta CD8 count: 202 vs. 102, *p* = 0.023; delta CD8 %: − 24 vs. − 13, *p* < 0.001), and larger CD4/CD8 ratio change at 48 months (delta CD4/CD8: 0.53 vs. 0.33, *p* < 0.001) (Table 1). These results suggest that different behaviors on 5′-LTR methylation could be associated with baseline clinical characteristics of persons starting ART.

**Fig. 5:**
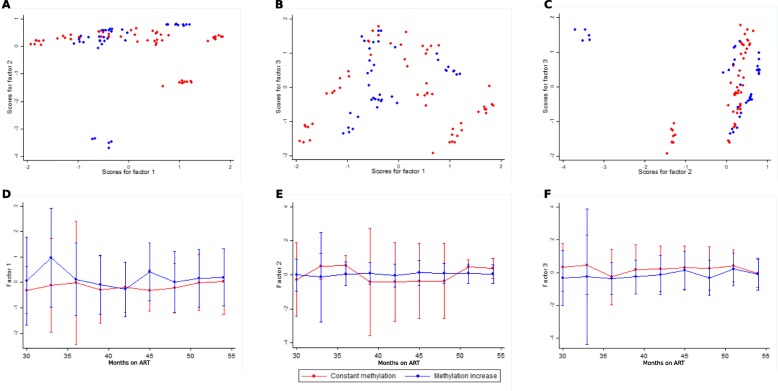
Principal component analysis. Factorial analysis using principal components with varimax rotation was used to describe data structure. **a**–**c** Factor scores for the three top factors that together explained 51% of the total variance are shown for each follow-up time-point for 12 participants on short-term ART. **d**–**f** Time trends for each of the three factors are shown. Color denotes participants classified in the constant methylation and the methylation increase groups

We applied factorial analysis using principal components (PCA) with varimax rotation in order to explain the structure of the data in an unbiased way. We included baseline and follow-up clinical variables (Fig. 5, see “Methods”). Altogether, the first three factors explained 51% of the total variance. Factor 1 explained 21% of the total variance and encompassed ten variables (age, CD4^+^ T cell number and % gain, CD8^+^ T cell number and % change, CD4/CD8 change, % CD4^+^ memory, namory, _CM_, and T_TM_ cells), factor 2 16% and encompassed seven variables (CD4^+^ T cell number gain, CD8^+^ T cell number change, time to undetectable pVL, time on ART: 30- vs. 54-months follow-up, third drug class in ART regimen: NNRTI vs. PI), and factor 3 13% and encompassed five variables (nadir CD4^+^ T cell count, CD8^+^ T cell count change, %T_EM_, %T_TE_, and number of ART regimen changes). Of note, CpG methylation index and pre-ART pVL were only included up to factor 4; ART regimen backbone in factor 5; and residual viral load, proviral load, and presence of blips were included up to factor 6. The constant methylation and methylation increase groups showed low evidence of significant separation along time (Wilcoxon test for factor 2: *p* = 0.04; factors 1 and 3: *p* > 0.05) (Fig. 5).

In all, these observations suggest that temporal differences in 5′-LTR methylation patterns could be associated with a combination of baseline and follow-up clinical characteristics, mainly age, CD4^+^ T cell gain, CD8^+^ T cell decrease, time on ART, class of third drug in ART regimen, and CD4 T cell subpopulation composition.

## Discussion

Many HIV cure efforts have focused on developing strategies to reverse viral latency that, in theory, could allow viral gene expression leading to virus recognition and eradication. The contribution of methylation of the HIV 5′-LTR promoter region to HIV latency remains elusive, with previous studies showing contrasting results. Our study shows for the first time that HIV 5′-LTR methylation is dynamic along time, with different methylation variation patterns possibly associated with baseline clinical characteristics of persons starting ART. These observations could explain some of the differences between previous studies showing contrasting results. Strengths and novel aspects of this study include implementation of a next generation sequencing-based methodology, able to extensively analyze the composition of 5′-LTR variants and their methylation status with high sensitivity, in contrast with previous studies using traditional clonal Sanger sequencing. Additionally, we present for the first time a longitudinal assessment of 5′-LTR methylation changes on CD4^+^ T cells of a cohort of individuals on short-term ART, and further compared these results with a cross-sectional group of persons with long-term ART. Our study shows that HIV 5′-LTR methylation in total blood CD4^+^ T cells is dynamic along time, and that at least two different methylation patterns exist that show association with both baseline and follow-up clinical characteristics of individuals, mainly age, CD4^+^ T cell gain, CD8^+^ T cell decrease, and CD4^+^ T cell subpopulation proportions.

It has been previously suggested that latent HIV resides mainly in resting CD4^+^ T cells [35, 36] and that DNA methylation may be a late event that reinforces silencing of the latent provirus [37, 38]. Indeed, CpG hypermethylation within the HIV 5′-LTR has been associated with silencing of the HIV promoter in latently infected Jurkat cells and resting CD4^+^ T cells from aviremic patients under ART [13, 14]. However, other studies analyzing the latent viral reservoir of aviremic patients on ART have found very low or no methylation on the HIV 5′-LTR in resting memory CD4^+^ T cells or peripheral blood mononuclear cells (PBMCs), questioning the importance of methylation as a maintenance mechanism of the latent reservoir [15, 17]. There are several factors that could account for contrasting observations on the role of 5′-LTR methylation on HIV latency, including the length of HIV infection, ART regimen used, time on ART, cell type studied, CD4^+^ T cell subpopulation composition, rate of replacement of latently infected CD4^+^ T cells, proportion of defective proviruses, and HIV integration sites [16, 17, 19]. Our study identified associations between several of these variables and distinct longitudinal methylation change patterns, including age, CD4^+^ T cell number and % gain, CD8^+^ T cell number and % change, proportions of CD4^+^ T cell subpopulations, third drug class in the current ART regimen, and time to undetectable viral load. Moreover, as recently suggested [39], other rather methodological factors could also account for differences and limit conclusions on methylation analyses including the use of cell lines versus primary CD4^+^ T cells in vitro, frozen versus fresh samples, the type of bisulfite treatment, sampling bias, polymerase chain reaction (PCR) amplification strategy and primers used, and sequencing technique (Sanger versus next generation sequencing). Using PCA, our study identified CD4^+^ T cell gain (at 48 months after ART initiation), CD8^+^ T cell change, CD4/CD8 rate change, and proportion of naïve, TCM, and TTM cells as the most important variables, accounting for 21% of the total variance in the study participants. Interestingly, time on ART, time to undetectable pVL, and third drug class in the ART regimen were also identified as important variables explaining total variance in the second eigenvector. Since DNA methylation could be a late event that strengthens the silencing of already latent viruses, rather than driving latency from the beginning, infection time and source cell type could be very important factors in methylation analyses. Indeed, CpG methylation may be a more common latency mechanism in memory CD4^+^ T cells that are more prone to proliferation than in resting CD4^+^ T cells [40]. Late selection of latent proviruses in the memory cell subpopulation could result in the accumulation of proviruses with methylated promoters as compared to unmethylated proviruses found in the resting cell subpopulation [40]. Recently, Trejbalova et al. showed a positive correlation between time on ART and the degree of CpG methylation in the HIV 5′-LTR in resting CD4+ T cells [19]. However, it is hard to discriminate if the correlation was due to time on ART only, time of HIV infection before ART initiation, or both. Our study contributes with additional information to the field, showing that 5′-LTR methylation appears early after ART start in at least two different patterns that can be associated with the baseline clinical characteristics of each person. Interestingly, while a group of participants maintained fairly high and constant methylation levels across the follow-up period, a significant increase in methylation burden occurred in a subset of participants (58%) at around 42 months (3.5 years) after ART initiation. This time point coincided with an overall significant decrease in proviral load, an observation that has been reported before in a large cohort [41]. The methylation increase subgroup had overall different baseline characteristics compared to the constant methylation subgroup as shown by PCA, and was composed mainly of younger individuals presenting late to clinical care (nadir CD4^+^ T cell count: 13 cells/mm^3^), and showing larger CD4^+^ cell gains. These observations could help to explain conflicting observations of previous studies and could be important for cure strategies.

Our study is unique in the sense that it used a highly effective bisulfite conversion system with low DNA degradation, a nested PCR strategy to selectively amplify and analyze bisulfite-converted 5′-LTR, and a highly sensitive next generation sequencing strategy that detects low frequency variants more efficiently than clonal Sanger sequencing strategies. As 5′-LTR methylation analyses were made on total CD4^+^ T cells, we accounted for the CD4^+^ T cell subpopulation composition in each sample as a possible confounder. Finally, our study design was longitudinal, which made it possible to study the dynamics of 5′-LTR methylation over time in the same individual.

With our experimental design, we were able to identify between 1 and 13 different variants (median: 3) per time point with median coverage of 16270 reads, and additionally estimate the prevalence of each variant within the sample, allowing to weight the contribution of each variant to the overall proviral landscape and methylation burden. This greatly exceeds the average number of clones analyzed per sample in other studies using Sanger-based sequencing strategies. Our controls showed a highly efficient bisulfite-conversion process (100% conversion), which combined with our primer design (that considered bisulfite-converted cytosines) (Additional file 5: Table S2) and filtering strategy, selected for PCR products derived from bisulfite-converted templates.

Some weaknesses of our study are worth mentioning: as we exclusively sequenced the 5′-LTR and not the complete HIV genome, we were not able to assess the role of 5′-LTR methylation in replication-competent proviruses only, nor the role of methylation in other parts of the provirus or the integration site. Moreover, our analyses were made in total CD4^+^ T cells due to limitations in cell number and the possibility of amplification of the LTR region in bisulfite-treated DNA, and thus, it was not possible to assess methylation in specific cell subpopulations or in resting CD4^+^ T cells. Also, as proviral loads were variable among participants and time points, even when the same amount of DNA was used for bisulfite conversion and as input for PCR amplification, the number of provirus templates analyzed in different samples was variable. Finally, as previously suggested [39], proviral load and residual pVL may not be the most appropriate outputs to assess the role of 5′-LTR methylation in latency as defective and replication-competent proviruses are both studied and cannot be differentiated in our method. Nevertheless, they are good proxy variables, given our high sampling depth, which significantly increases sensitivity to include intact proviruses. Indeed, considering our median proviral load (8129 copies/million CD4^+^ cells), when starting with 900 ng of DNA for bisulfite conversion, we would be working with 136,364 human genomes, from which 1109 would contain HIV DNA. If we consider that 88% of proviruses are defective [7], we would have a total of 133 intact proviruses in the sample. As half of the volume of bisulfite-converted DNA was used for PCR, we would be analyzing 67 intact proviruses (without considering inherent DNA degradation due to bisulfite treatment). Furthermore, this study did not contemplate large blood draws or collection of leukapheresis samples, necessary to perform viral outgrowth assays [5, 42], given the low proportion of cells expected to contain latent HIV in blood in vivo (1 in 10^6^ resting CD4^+^ T cells) [5, 43] in comparison with in vitro latency models, and thus it was not possible to perform virus reactivation analyses.

Further insight on the observed alternation between unmethylated and methylated variants is warranted to understand the overall role of 5′-LTR methylation on HIV latency. This could be a complex phenomenon with several possible mechanisms in play. Regarding absence of or low-level 5′-LTR methylation in cells with latent infection or low-level HIV expression, important mechanisms could include (1) prevention of de novo methylation [18, 44], (2) high rate of demethylation [44], or (3) deamination of 5mCpG or CpG (detection of a TpG site may be the result of deamination of either 5mCpG or unmethylated CpG, both of which would produce UpG, which would in turn be observed as TpG after PCR amplification). Indeed, it has been suggested that methylation could be the main mechanism responsible for depletion of CpG sites in the HIV genome [45]. This type of mutation could either originate replication-defective proviruses or remove transcriptional silencing [46]. Regarding enrichment of methylated variants, some possible mechanisms include (1) accumulation of proviruses with methylated promoters in memory cells through homeostatic proliferation [47, 48], (2) change in the proportion of the cellular subpopulations containing proviral DNA [18], (3) de novo methylation [19, 49], and (4) low-level cellular activation.

## Conclusions

We show strong evidence that HIV 5′-LTR methylation can vary significantly along time in proviruses from circulating CD4^+^ T cells and that methylation is observed from early stages of ART initiation. We also show that 5′-LTR methylation can follow different temporal patterns that are associated with baseline clinical characteristics, and which may account for some differences observed between previous contrasting studies. In particular, different 5′-LTR methylation patterns can be associated with age, CD4^+^ T cell gain and CD8^+^ T cell change after ART initiation, changes in proportions of circulating T cell subpopulations (mainly T_CM_, T_TM_, and naïve), time on ART, time to undetectable pVL, and third drug class in the ART regimen.

Further studies in specific cell subpopulations and in tissues, the use of larger cohorts with longer follow-up times and stricter control of confounding variables (e.g., infection time) and the use of novel sequencing techniques, allowing assessment of methylation along the complete provirus and in the integration site are necessary to assess the role of viral LTR methylation in proviral silencing and the extent to which it might be clinically relevant.

## Methods

### Ethics statement

This study was approved by the Institutional Review Board of the National Institute of Respiratory Diseases (INER) (registry: DHHS IORG0003948; CONBIOETICA-09-CEI-003-20160427) in Mexico City (project code: B26-09), and was conducted according to the principles of the Declaration of Helsinki. All participants gave written informed consent before blood sample donation.

### Patients

This study included two parallel designs, longitudinal and cross-sectional. Longitudinal blood samples from 12 HIV-infected persons with a short history of suppressive ART (21–30 months on ART and viral load 40 copies/mL) were obtained in -month intervals for 2 years. Cross-sectional blood samples from ten additional HIV-infected persons with a long history of suppressive ART (6–15 years) were obtained. All blood samples were obtained and processed at the Center for Research in Infectious Diseases (CIENI) of INER in Mexico City. Plasma and DNA of total CD4^+^ T cells were stored from each blood sample donation. Plasma viral loads for clinical follow-up were determined using the m2000 system (Abbott, Abbott Park, IL, USA). CD4^+^ and CD8^+^ T cell counts were assessed by flow cytometry using the TruCount kit in a FACSCanto II instrument (BD Bioscience, San Jose, CA, USA).

### Residual plasma viral load measurement

Viral RNA was extracted from 9 mL of plasma. Plasma was centrifuged at 15,000 rpm for 3 h, at 4 °C. The supernatant was removed and the pellet re-suspended in 140 µL. Viral RNA was purified from the pellet using QIAamp viral RNA mini kit (QIAGEN, Valencia, CA, USA; resuspending in 41 µL of elution buffer) and stored at -80 °C until use. Residual plasma viral load (<40 RNA copies/mL)was determined by real-time PCR (qPCR) with TaqMan Fast Virus One-Step Master Mix (ThermoFisher, Waltham, MA, USA) using an in-house NL4-3-derived RNA standard. NL4-3 RNA was extracted from culture supernatants (QIAamp viral RNA mini kit, QIAGEN) and quantified (m2000 system, Abbott). The standard curve was generated performing two-fold dilutions ranging from 256 to 1 RNA copies. qPCR was performed in triplicate for standard curve points and duplicate for sample points using primers Fwd 5′-GGTCTCTCTGGTTAGACCAGAT-3′ (HXB2 positions: 455–476), Rv 5′-CTGCTAGAGATTTTCCACACTG-3′ (635–614), and the TaqMan probe 5′-6FAM-AGTAGTGTGTGCCCGTCTGTT-TAMRA-3′ (552–572), directed to the viral LTR, in 50 µL reactions containing 20.5 μL of standard or sample RNA, on a 7500 Real Time PCR System instrument (ThermoFisher). qPCR conditions were as follows: 50 °C for 50 min for reverse transcription, 95 °C for 20 s, followed by 40 cycles of 95 °C for 15 s and 60 °C for 60 s.

### Proviral load measurement from total CD4^+^ cells

Peripheral blood mononuclear cells (PBMC) were obtained from 16 mL of blood by Ficoll gradient centrifugation. Total CD4^+^ T cells were purified by negative selection from 50 million PBMC, using EasySep Human CD4^+^ T Cell kit (StemCell Technologies, Vancouver, Canada), and DNA was purified using iPrep Purelink gDNA Blood kit (ThermoFisher) on an iPrep Nucleic Acid Purification System (ThermoFisher). Proviral load was measured from total CD4^+^ T cells using TaqMan Gene Expression Master Mix (ThermoFisher). An in-house DNA standard derived from the ACH-2 cell line (obtained through the National Institutes of Health AIDS Reagent Program; catalogue 349) [50], containing two HIV proviral copies per cell, was used [51, 52]. To construct the standard curve, DNA was extracted from 1 × 10^6^ ACH-2 cells (iPrep Purelink gDNA Blood kit; ThermoFisher), and quantified (NanoDrop 1000; Thermo Scientific). The standard curve was obtained from 12 1:3 serial dilution points starting from a DNA stock containing 400 ng, in triplicate. qPCR was performed from 400 ng total CD4^+^ T cell DNA, using the same primers and probe used for residual plasma viral load quantification (see above). RNase P quantification was used as internal reference (Taqman RNase P Control Reagents kit; ThermoFisher). PCR conditions were as follows: 95 °C for 10 min, followed by 45 cycles of 95 °C for 15 s and 60 °C for 1 min. All values were corrected for CD4^+^ T cell purity, assessed by multi-color flow cytometry as explained below, and adjusted to 1 × 10^6^ genomes.

### Viral LTR methylation analysis method

#### Bisulfite conversion

Methylation was assessed by bisulfite conversion using 900 ng of total CD4^+^ T cell DNA, with EpiTect Fast DNA Bisulfite kit (Qiagen), on a QIAcube instrument (Qiagen). Samples were eluted in 16 μL elution buffer. The complete volume of bisulfite-treated DNA was used immediately to perform first-round LTR-Gag PCR in duplicate, as described below. Human Methylated & Non-methylated DNA Set kit (Zymo Reseach, Irvine, CA, USA) was used as control to evaluate the efficiency of bisulfite conversion, according to manufacturer’s specifications, using Platinum Taq DNA polymerase (ThermoFisher). Illumina adaptors for sequencing library construction were added to the primers recommended by the manufacturer (Additional file 5: Table S2).

#### 5′-LTR amplification

The viral 5′-LTR region was amplified using nested PCR, with Platinum Taq DNA polymerase (ThermoFisher), obtaining U3-R amplicons from both bisulfite-treated and untreated samples. Illumina adaptors for sequencing library construction were included as part of the second-round primers (Additional file 5: Table S2). For bisulfite-untreated samples, primers LTR1-20Fw and LTR835-856Rv (Additional file 5: Table S2) were used for the first-round PCR, allowing amplification of an LTR-*gag* fragment, with the following conditions: 94 °C for 3 min, 35 cycles of 94 °C for 30 s, 50 °C for 30 s, and 72 °C for 1 min, and a final step of 72 °C for 5 min. For the second-round PCR, primers Nex-LTR213-236Fw and Nex-LTR471-495Rv were used, allowing amplification of the U3-R region of the 5′-LTR, with the following conditions: 94 °C for 3 min, 35 cycles of 94 °C for 30 s, 56 °C for 30 s, and 72 °C for 1 min, and a final step of 72 °C for5 min. For bisulfite-treated samples, primers LTR1-20TxFw and LTR835-856TxRv were used for the first-round PCR with the following conditions: 94 °C for 3 min, 35 cycles of 94 °C for 30 s, 48 °C for 30 s, and 72 °C for 1 min, and a final step of 72 °in, an5 min. For the second-round PCR, primers Nex-LTR213-236TxFw and Nex-LTR471-495TxRv were used, with the following conditions: 94 °C for 3 min, 35 cycles of 94 °C for 30 s, 47 °C for 30 s, and 72 °C for 1 min, and a final step of 72 °C for5 min.

#### LTR next generation sequencing

Next generation sequencing (NGS) libraries were constructed from purified U3-R LTR amplicons (both from bisulfite-treated and -untreated DNA), using the amplicon approach with Nextera XT DNA sample preparation and index kits (Illumina, San Diego, CA, USA) (Fig. 1). Unique index combinations were added to each sample for multiplexing. Libraries were normalized to 60 nM and agarose gel-purified (338–440 bp bands, QIAquick gel extraction kit, Qiagen). After normalizing again, libraries were pooled, quantified (Qubit 3.0 Fluorometer; ThermoFisher), and run on a MiSeq instrument (Illumina), using v2 300-cycle pair-end kits (Illumina), according to manufacturer’s instructions.

#### Read alignment and filtering

Reads were assembled and filtered using USEARCH program version 9.2.64 [53]. The following algorithm was used: (1) fastq R1 and R2 files were fused together, (2) reads were filtered by primer binding site presence (allowing a maximum of two nucleotide difference) and size (209–309 nucleotides for samples, 224–324 nucleotides for the DAPK methylation controls), (3) primer sequences were removed (24 and 25 nucleotides at the 5′ and 3′ end respectively for the samples, 32 and 35 for the controls), (4) reads were filtered by quality (maximum expected error = 1), (5) abundance of each sequence was determined, (6) singlets were eliminated, and (7) reads with abundance < 2% were filtered out. The reads obtained from USEARCH software showed 97.6% (range 95.1–100%) conversion rate of cytosines outside CpG sites for the samples and 100% for the controls, using the software Quma (Quantification Tool for Methylation Analysis) [54].

### LTR methylation analysis calculations

Sequencing reads obtained for each time point for each individual were normalized to 100 and aligned to the HXB2 reference with Clustal Omega [55]. A position-specific score matrix (PSSM) was constructed using R and Seqinr [56]. The nine canonical CpG sites in the amplified HIV LTR region were located in the PSSM and the proportion of methylated (CpG), non-methylated (TpG), and mutated sequences was estimated for each one. The PSSM also provided information about additional CpG, CHG, and CHH methylation sites. These data were represented by multiple pie graphs using the program Raw Graphs [57].

We used Quma software [54], optimized for NGS reads analysis, to quantify and evaluate differences in global methylation, and estimated a “CpG Methylation Index” metric, averaging the proportion of methylated sequences for each of the nine canonical HXB2 LTR CpG sites in each sample. The CpG Methylation Index metric ranged from 0 (completely unmethylated) to 100 (fully methylated). Methylation differences between samples of different time-points were assessed using Mann-Whitney U test, using Quma. In order to be more comprehensive in the methylation analysis, we also studied non-canonical methylation patterns along the follow-up period, establishing a measure of methylation on CpG sites different to the canonical ones, present in the HXB2 reference (additional CpG methylation index), and methylation in sites other than CpG (non-CpG methylation index), averaging the proportion of methylated sequences for each relevant position in each category.

### Immunophenotyping

A panel of 12 antibodies was selected to identify by flow cytometry CD4^+^ T lymphocyte subpopulation proportions from each sample time point for all participants included in the study. Antibodies used included the following: *Biolegend*: HLA-DR BV785 clone L243 (307641), CD45RO BV650 clone UCHL1 (304231), CD3 BV 570 clone UCHT1 (300435), CD95 PECy5 clone DX2 (305610), CCR7 APC Cy7 clone G043H7 (353211), CCR5 BV421 clone J418F1 (359117), PD1 PE clone EH12.2H7 (329906), and CD127 FITC clone A019D5 (351311). *BD*: CD28 BV711 clone CD28.2 (563131), CD38 AF700 clone HIT2 (560676), CD45 APC clone RUO (340943). *Invitrogen*: CD4 PE Cy5.5 clone S3.5 (MHCD0418). *Life technologies*: LIVE/DEAD Fixable Aqua Dead Cell Stain Kit (L34957).

All antibodies were previously titrated using healthy donor CD4^+^ T lymphocytes. For staining, 1 × 10^6^ CD4^+^ T cells were resuspended in 2 mL of staining buffer (1× PBS, 2% fetal bovine serum and 0.5% EDTA). Cells were centrifuged at 2000 rpm for 5 min and decanted. Staining was carried out in the remaining volume (approximately 50 μL), adding the anti-CCR7 antibody first. After 10-min incubation,the rest of the antibodies were subsequently added and the tube was incubated for 1 h at room temperature in the dark. Cells were then washed with 3 mL of staining buffer, centrifuged at 2000 rpm for 5 min, decanted, and fixed with 0.3 mL of 1% paraformaldehyde. Cells were acquired on an LSR Fortessa cytometer (BD).

CD4^+^ T cell subpopulations were defined as follows: Naïve CD4^+^ T cells (CD3^+^/CD4^+^/CD45RO^−^/CCR7^+^/CD28^+^/CD95^−^), memory CD4^+^ T cells (CD3^+^/CD4^+^/CD28^+ −^/CD95^+^), stem cell memory (T_SCM_: CD3^+^/CD4^+^/CD45RO^−^/CCR7^+^/CD28^+^/CD95^+^), central memory (T_CM_: CD3^+^/CD4^+^/CD45RO^+^/CCR7^+^/CD28^+^), transitional memory (T_TM_: CD3^+^/CD4^+^/CD45RO^+^/CCR7^−^/CD28^+^), effector memory (T_EM_: CD3^+^/CD4^+^/CD45RO^+^/CCR7^−^/CD28^−^), terminal effector (T_TE_: CD3^+^/CD4^+^/CD45RO^−^/CCR7^−^/CD28^−^), and T new (T_NEW_: CD3^+^/CD4^+^/CD45RO^−^/CCR7^−^/CD28^+^) using the program Cytobank [58]. For cell subpopulations with a limited number of captured events, we used a Poisson distribution to establish the minimal threshold for analysis in 100 positive events and 10% variation coefficient. The gating strategy is shown in Additional file 3: Figure S2.

### Statistical analysis

Comparisons of clinical and methylation parameters of the longitudinal data were performed for each time-point against month 30 as reference (overall and both for the methylation increase and constant methylation groups), and against the long-term ART group (months 30 and 42 are shown in figures and tables). Medians with interquartile ranges were used for descriptive analysis. Variables from related samples (different time-points form the same participant) were compared using the Wilcoxon test and variables from independent samples were compared using the Mann-Whitney test. A *p* < 0.05 was considered significant. Statistical analyses were conducted using SPSS version 23.0 and STATA version 14.0.

Exploratory factorial analysis using principal components (PCA) was used as a variable reduction method to describe data structure and summarize the variance in baseline and follow-up clinical variables into fewer dimensions. Indexes were constructed, followed by orthogonal (varimax) rotation. Variables included were age, nadir CD4^+^ T cell count, pre-ART pVL, delta CD4^+^ T cell count and % at 48 months, delta CD8^+^ T cell count and % at 48 months, CD4/CD8 ratio change at 48 months, time on ART, time to achieve undetectable pVL, residual pVL, proviral load, CpG methylation index, number of ART regimen changes, ART backbone (tenofovir + emtricitabine vs. abacavir + lamivudine), ART third drug (non-nucleoside RT inhibitors vs. protease inhibitors), presence of blips, and CD4^+^ T cell subpopulation proportions: naeoside RT inhibitors v_SCM_, T_CM_, T_EM_, T_TM_, T_NEW_, T_TE_. Six factors showed an eigenvector value higher than 1 (cumulative variance 77%), from which three were selected (cumulative variance 51%) for further presentation and analysis. Factor scores were obtained and graphed. PCA was performed using STATA version 14.0.

## Additional files

Additional file 1: **Figure S1.** Gating strategy to define CD4+ T cell subpopulations. (PDF 529 kb)

Additional file 2: **Table S1.** Antiretroviral treatment history of participants. (DOC 54 kb)

Additional file 3: **Figure S2.** Detailed HIV 5’-LTR variant composition and methylation analysis in the rest of participants. Detailed variant composition per time point is shown for ten participants with short-term antiretroviral treatment not included in Fig. 3 (TP23, TP24, TP27, TP29, TP30, TP32, TP26, TP28, TP33, TP34), and for the single time point available for the 10 participants with long-term antiretroviral therapy (TP35, TP36, TP37, TP38, TP40, TP41, TP43, TP44, TP45, TP46). Each horizontal line represents a variant. The number of reads representing that variant and its proportion in the sample are shown on the right. Each circle represents a canonical CpG site, numbered according to the HXB2 reference: black–methylated, white–unmethylated, gray–mutated. (PDF 16804 kb)

Additional file 4: **Figure S3.** Changes in 5’-LTR overall methylation patterns along time. A comparison of methylation patterns is shown for all participants with short-term antiretroviral therapy (ART) at 30 months (2.5 years) of follow-up (top), all participants with short-term ART at 42 months (3.5 years) of follow-up (center) and all participants with long-term ART (single time point available, 6 to 15 years) (bottom). Each line depicts a summary of all variants observed per time point. CpG sites are shown as pie charts indicating the proportion of methylated (black), unmethylated (light gray) or mutated (dark gray) variants in the sample. Canonical HXB2 CpG positions are numbered. Additional CpG, CHG and CHH methylation-susceptible sites, not observed in the HXB2 sequence, are also included (marked with *). The number of reads and the total number of variants obtained per time point, after filtering and aligning, are shown. Additionally, measurements of residual plasma viral load, proviral load and proportion of memory (CD3+/CD4+/CD28+−/CD95+; black) and naïve (CD3+/CD4+/CD45RO-/CCR7+/CD28+/CD95-; light gray) CD4+ T cells per time point are shown. (PDF 207 kb)

Additional file 5: **Table S2.** Primers for HIV 5’-LTR and methylation control amplification and sequencing. (DOCX 17 kb)

## Data Availability

The datasets used during the current study are available from the corresponding author on reasonable request.

## Abbreviations

ART: Antiretroviral treatment
DAPK1: Death-associated protein kinase 1
HDACi: Histone deacetylase inhibitor
HIV: Human immunodeficiency virus
IQR: Interquartile range
LTR: Long terminal repeat
NGS: Next generation sequencing
NNRTI: Non-nucleoside reverse transcriptase inhibitors
NRTI: Nucleoside reverse transcriptase inhibitors
PBMC: Peripheral blood mononuclear cells
PCR: Polymerase chain reaction
PI: Protease inhibitors
PSSM: Position-specific score matrix
pVL: Plasma viral load
T_CM_: Central memory T cells
T_EM_: Effector memory T cells
T_NEW_: T new
T_SCM_: Stem cell memory T cells
T_TE_: Terminally differentiated effector T cells
T_TM_: Transitional memory T cells

## References

[1] Flexner C, Plumley B, Brown Ripin DH (2013). Treatment optimization: an outline for future success. Curr Opin HIV AIDS..

[2] Furtado MR, Callaway DS, Phair JP, Kunstman KJ, Stanton JL, Macken CA (1999). Persistence of HIV-1 transcription in peripheral-blood mononuclear cells in patients receiving potent antiretroviral therapy. N Engl J Med..

[3] Chun TW, Stuyver L, Mizell SB, Ehler LA, Mican JA, Baseler M (1997). Presence of an inducible HIV-1 latent reservoir during highly active antiretroviral therapy. Proc Natl Acad Sci U S A..

[4] Chun TW, Engel D, Mizell SB, Hallahan CW, Fischette M, Park S (1999). Effect of interleukin-2 on the pool of latently infected, resting CD4+ T cells in HIV-1-infected patients receiving highly active anti-retroviral therapy. Nat Med..

[5] Eriksson S, Graf EH, Dahl V, Strain MC, Yukl SA, Lysenko ES (2013). Comparative analysis of measures of viral reservoirs in HIV-1 eradication studies. PLoS Pathog..

[6] Cillo AR, Sobolewski MD, Bosch RJ, Fyne E, Piatak M, Coffin JM (2014). Quantification of HIV-1 latency reversal in resting CD4+ T cells from patients on suppressive antiretroviral therapy. Proc Natl Acad Sci U S A..

[7] Ho YC, Shan L, Hosmane NN, Wang J, Laskey SB, Rosenbloom DI (2013). Replication-competent noninduced proviruses in the latent reservoir increase barrier to HIV-1 cure. Cell..

[8] Rasmussen TA, Tolstrup M, Brinkmann CR, Olesen R, Erikstrup C, Solomon A (2014). Panobinostat, a histone deacetylase inhibitor, for latent-virus reactivation in HIV-infected patients on suppressive antiretroviral therapy: a phase 1/2, single group, clinical trial. The Lancet HIV..

[9] Du Chene I, Basyuk E, Lin YL, Triboulet R, Knezevich A, Chable-Bessia C (2007). Suv39H1 and HP1gamma are responsible for chromatin-mediated HIV-1 transcriptional silencing and post-integration latency. EMBO J..

[10] Keedy KS, Archin NM, Gates AT, Espeseth A, Hazuda DJ, Margolis DM (2009). A limited group of class I histone deacetylases acts to repress human immunodeficiency virus type 1 expression. J Virol..

[11] Imai K, Togami H, Okamoto T (2010). Involvement of histone H3 lysine 9 (H3K9) methyltransferase G9a in the maintenance of HIV-1 latency and its reactivation by BIX01294. J Biol Chem..

[12] Friedman J, Cho WK, Chu CK, Keedy KS, Archin NM, Margolis DM (2011). Epigenetic silencing of HIV-1 by the histone H3 lysine 27 methyltransferase enhancer of Zeste 2. J Virol..

[13] Blazkova J, Trejbalova K, Gondois-Rey F, Halfon P, Philibert P, Guiguen A (2009). CpG methylation controls reactivation of HIV from latency. PLoS Pathog..

[14] Kauder SE, Bosque A, Lindqvist A, Planelles V, Verdin E (2009). Epigenetic regulation of HIV-1 latency by cytosine methylation. PLoS Pathog..

[15] Palacios JA, Perez-Pinar T, Toro C, Sanz-Minguela B, Moreno V, Valencia E (2012). Long-term nonprogressor and elite controller patients who control viremia have a higher percentage of methylation in their HIV-1 proviral promoters than aviremic patients receiving highly active antiretroviral therapy. J Virol..

[16] Doerfler W, Weber S, Kemal K, Weiser B, Korn K, Anastos K (2012). Epigenetic modifications of HIV proviral LTRs: potential targets for cure. Retrovirology..

[17] Blazkova J, Murray D, Justement JS, Funk EK, Nelson AK, Moir S (2012). Paucity of HIV DNA methylation in latently infected, resting CD4+ T cells from infected individuals receiving antiretroviral therapy. J Virol..

[18] Weber S, Weiser B, Kemal KS, Burger H, Ramirez CM, Korn K (2014). Epigenetic analysis of HIV-1 proviral genomes from infected individuals: predominance of unmethylated CpG's. Virology..

[19] Trejbalova K, Kovarova D, Blazkova J, Machala L, Jilich D, Weber J (2016). Development of 5' LTR DNA methylation of latent HIV-1 provirus in cell line models and in long-term-infected individuals. Clin Epigenetics..

[20] Yoon CH, Jang DH, Kim KC, Park SY, Kim HY, Kim SS (2014). Disruption of polycomb repressor complex-mediated gene silencing reactivates HIV-1 provirus in latently infected cells. Intervirology..

[21] Matsuda Y, Kobayashi-Ishihara M, Fujikawa D, Ishida T, Watanabe T, Yamagishi M (2015). Epigenetic heterogeneity in HIV-1 latency establishment. Sci Rep..

[22] Rafati H, Parra M, Hakre S, Moshkin Y, Verdin E, Mahmoudi T (2011). Repressive LTR nucleosome positioning by the BAF complex is required for HIV latency. PLoS Biol..

[23] Van Duyne R, Guendel I, Narayanan A, Gregg E, Shafagati N, Tyagi M (2011). Varying modulation of HIV-1 LTR activity by Baf complexes. J Mol Biol..

[24] Harwig A, Das AT, Berkhout B (2014). Retroviral microRNAs. Curr Opin Virol..

[25] Patel P, Ansari MY, Bapat S, Thakar M, Gangakhedkar R, Jameel S (2014). The microRNA miR-29a is associated with human immunodeficiency virus latency. Retrovirology..

[26] Barichievy S, Naidoo J, Mhlanga MM (2015). Non-coding RNAs and HIV: viral manipulation of host dark matter to shape the cellular environment. Front Genet..

[27] Rice AP (2015). Roles of microRNAs and long-noncoding RNAs in human immunodeficiency virus replication. Wiley Interdiscip Rev RNA..

[28] Suzuki K, Ahlenstiel C, Marks K, Kelleher AD (2015). Promoter targeting RNAs: unexpected contributors to the control of HIV-1 transcription. Mol Ther Nucleic Acids..

[29] Li E (2002). Chromatin modification and epigenetic reprogramming in mammalian development. Nat Rev Genet..

[30] Christman JK, Weich N, Schoenbrun B, Schneiderman N, Acs G (1980). Hypomethylation of DNA during differentiation of Friend erythroleukemia cells. J Cell Biol..

[31] Koiwa T, Hamano-Usami A, Ishida T, Okayama A, Yamaguchi K, Kamihira S (2002). 5'-Long terminal repeat-selective CpG methylation of latent human T-cell leukemia virus type 1 provirus In Vitro and In Vivo. Journal of Virology..

[32] Harbers K, Schnieke A, Stuhlmann H, Jahner D, Jaenisch R (1981). DNA methylation and gene expression: endogenous retroviral genome becomes infectious after molecular cloning. Proc Natl Acad Sci U S A..

[33] Hejnar J, Plachy J, Geryk J, Machon O, Trejbalova K, Guntaka RV (1999). Inhibition of the rous sarcoma virus long terminal repeat-driven transcription by in vitro methylation: different sensitivity in permissive chicken cells versus mammalian cells. Virology..

[34] Lavie L, Kitova M, Maldener E, Meese E, Mayer J (2005). CpG methylation directly regulates transcriptional activity of the human endogenous retrovirus family HERV-K (HML-2). J Virol..

[35] Persaud D, Pierson T, Ruff C, Finzi D, Chadwick KR, Margolick JB (2000). A stable latent reservoir for HIV-1 in resting CD4(+) T lymphocytes in infected children. J Clin Invest..

[36] Trono D, Van Lint C, Rouzioux C, Verdin E, Barre-Sinoussi F, Chun TW (2010). HIV persistence and the prospect of long-term drug-free remissions for HIV-infected individuals. Science..

[37] Duverger A, Jones J, May J, Bibollet-Ruche F, Wagner FA, Cron RQ (2009). Determinants of the establishment of human immunodeficiency virus type 1 latency. J Virol..

[38] Karn J (2011). The molecular biology of HIV latency: breaking and restoring the Tat-dependent transcriptional circuit. Curr Opin HIV AIDS..

[39] LaMere SA, Chaillon A, Huynh C, Smith DM, Gianella S. Challenges in quantifying cytosine methylation in the HIV provirus. MBio. 2019;10. 10.1128/mBio.02268-18 PMC6343035 30670613

[40] Van Lint C, Bouchat S, Marcello A (2013). HIV-1 transcription and latency: an update. Retrovirology..

[41] Bachmann N, Von Siebenthal C, Vongrad V, Neumann K, Turk T, Beerenwinkel N, et al. Determinants of HIV-1 reservoir size and long-term dynamics under suppressive ART In: 25th CROI Conference on Retroviruses and Opportunistic Infections; 2018 March 4–7; Boston, Massachusetts. United States of America; 2018. Available from: http://www.croiconference.org/sessions/determinants-hiv-1-reservoir-size-and-long-term-dynamics-under-suppressive-art.

[42] Huang SH, Ren Y, Thomas AS, Chan D, Mueller S, Ward AR (2018). Latent HIV reservoirs exhibit inherent resistance to elimination by CD8+ T cells. J Clin Invest..

[43] Finzi D, Hermankova M, Pierson T, Carruth LM, Buck C, Chaisson RE (1997). Identification of a reservoir for HIV-1 in patients on highly active antiretroviral therapy. Science..

[44] Pion M, Jordan A, Biancotto A, Dequiedt F, Gondois-Rey F, Rondeau S (2003). Transcriptional suppression of in vitro-integrated human immunodeficiency virus type 1 does not correlate with proviral DNA methylation. J Virol..

[45] Alinejad-Rokny H, Anwar F, Waters SA, Davenport MP, Ebrahimi D (2016). Source of CpG depletion in the HIV-1 genome. Mol Biol Evol..

[46] Hernando-Herraez I, Garcia-Perez R, Sharp AJ, Marques-Bonet T (2015). DNA Methylation: insights into human evolution. PLoS Genet..

[47] Chomont N, El-Far M, Ancuta P, Trautmann L, Procopio FA, Yassine-Diab B (2009). HIV reservoir size and persistence are driven by T cell survival and homeostatic proliferation. Nat Med..

[48] Von Stockenstrom S, Odevall L, Lee E, Sinclair E, Bacchetti P, Killian M (2015). Longitudinal genetic characterization reveals that cell proliferation maintains a persistent HIV type 1 DNA pool during effective HIV therapy. J Infect Dis..

[49] Rosca A, Anton G, Ene L, Iancu I, Temereanca A, Achim CL (2017). Immunoassay and molecular methods to investigate DNA methylation changes in peripheral blood mononuclear cells in HIV infected patients on cART. J Immunoassay Immunochem..

[50] Clouse KA, Powell D, Washington I, Poli G, Strebel K, Farrar W (1989). Monokine regulation of human immunodeficiency virus-1 expression in a chronically infected human T cell clone. J Immunol..

[51] Cannon P, Kim SH, Ulich C, Kim S. Analysis of Tat function in human immunodeficiency virus type 1-infected low-level-expression cell lines U1 and ACH-2. J Virol. 1994;68:1993-7. 10.1128/jvi.68.3.1993-1997.1994 PMC236665 8107261

[52] Symons J, Chopra A, Malatinkova E, De Spiegelaere W, Leary S, Cooper D (2017). HIV integration sites in latently infected cell lines: evidence of ongoing replication. Retrovirology..

[53] Edgar RC, Flyvbjerg H (2015). Error filtering, pair assembly and error correction for next-generation sequencing reads. Bioinformatics..

[54] Kumaki Y, Okano M: QUMA (QUantification tool for Methylation Analysis). http://quma.cdb.riken.jp (2019). Accessed 13 Feb 2019. 10.1093/nar/gkn294 PMC2447804 18487274

[55] EMBL-EBI: Clustal Omega. https://www.ebi.ac.uk/Tools/msa/clustalo/ (2018). Accessed 7 Apr 2018.

[56] Charif D, Lobry JR, Bastolla U, Porto M, Roman E, Vendruscolo M (2007). SeqinR 1.0-2: A contributed package to the R project for statistical computing devoted to biological sequences retrieval and analysis. Structural approaches to sequence evolution: Molecules, networks, populations.

[57] Mauri M, Elli T, Caviglia G, Uboldi G, Azzi M (2017). RAWGraphs: a visualisation platform to create open outputs. Proceedings of CHItaly '17, Cagliari, Italy.

[58] Kotecha N, Krutzik PO, Irish JM (2010). Web-based analysis and publication of flow cytometry experiments. Curr Protoc Cytom..

