# Supplementary figures and images for: Longitudinal variation in human immunodeficiency virus long terminal repeat methylation in individuals on suppressive antiretroviral therapy
Source: Clin Epigenetics. 2019 Sep 13;11:134. doi: 10.1186/s13148-019-0735-9 (PMC6743183; doi:10.1186/s13148-019-0735-9)

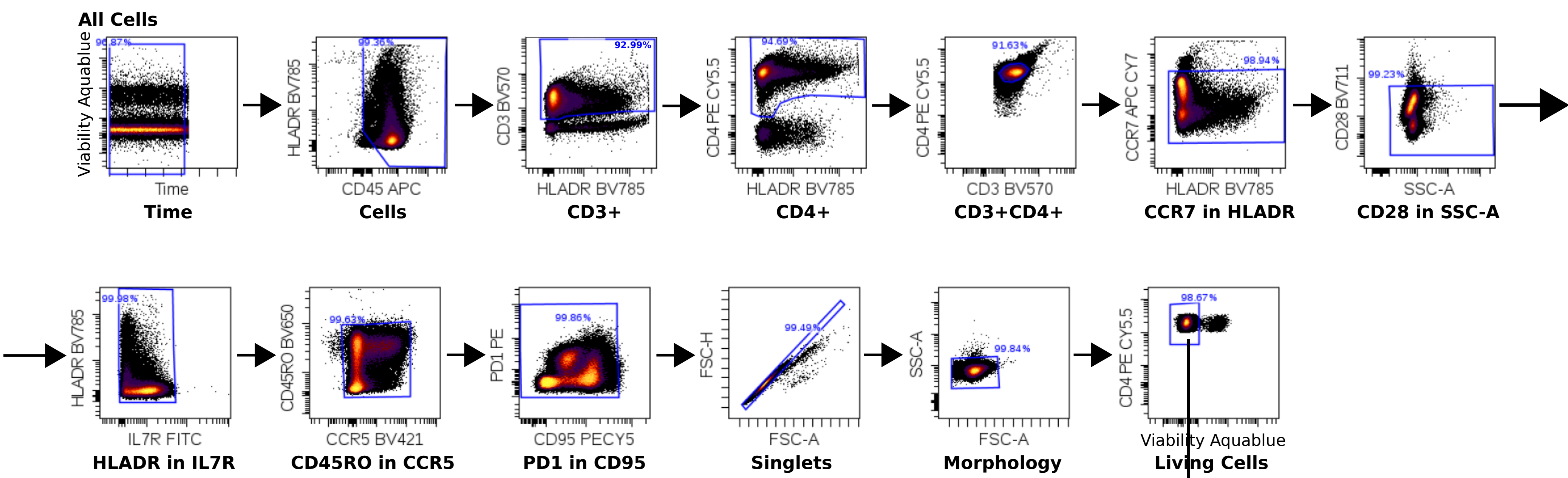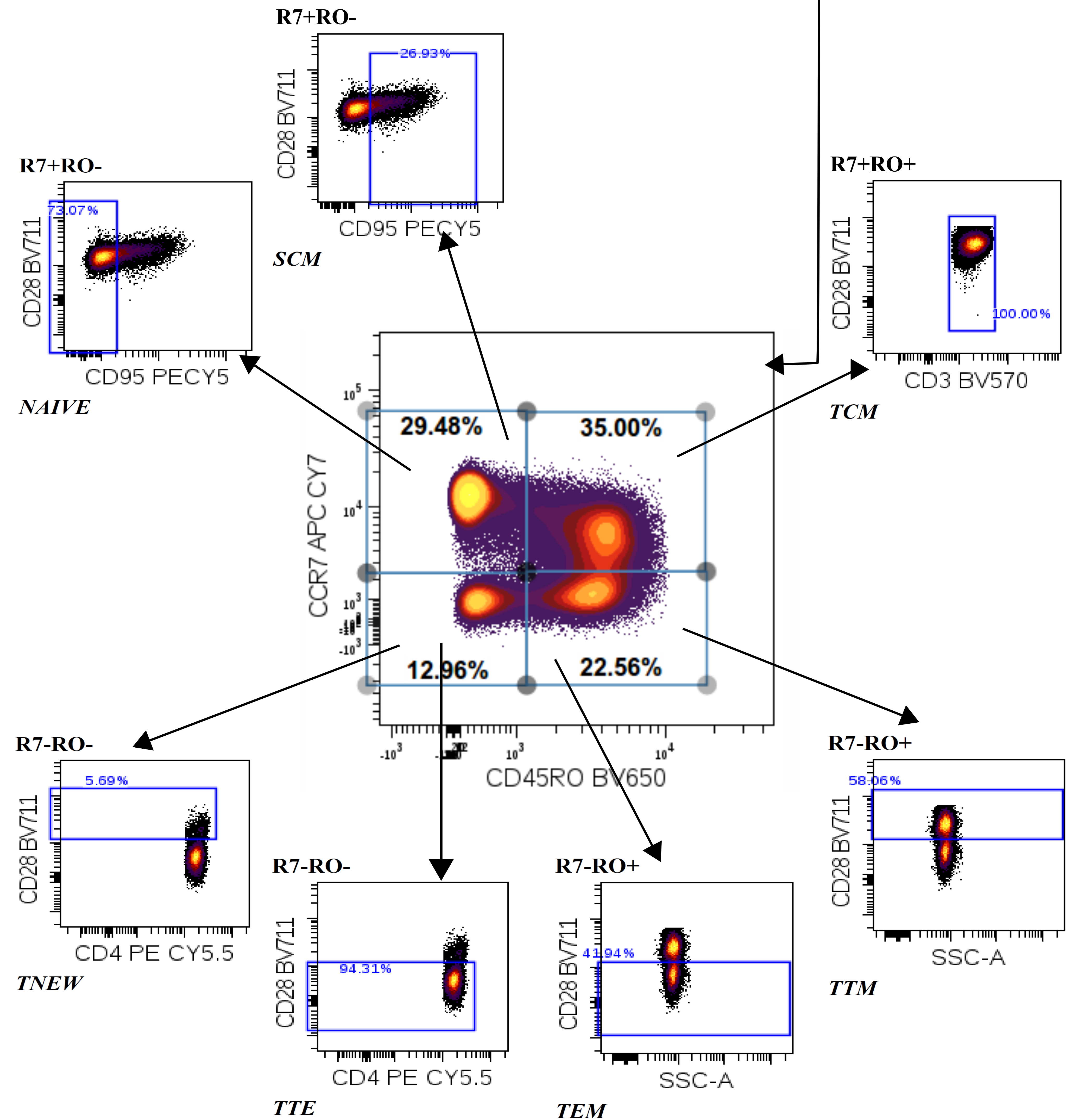

Supplement: Supplementary file 1 — Figure S1. Gating strategy to define CD4+ T cell subpopulations. (PDF 529 kb) [file 13148_2019_735_MOESM1_ESM.pdf]

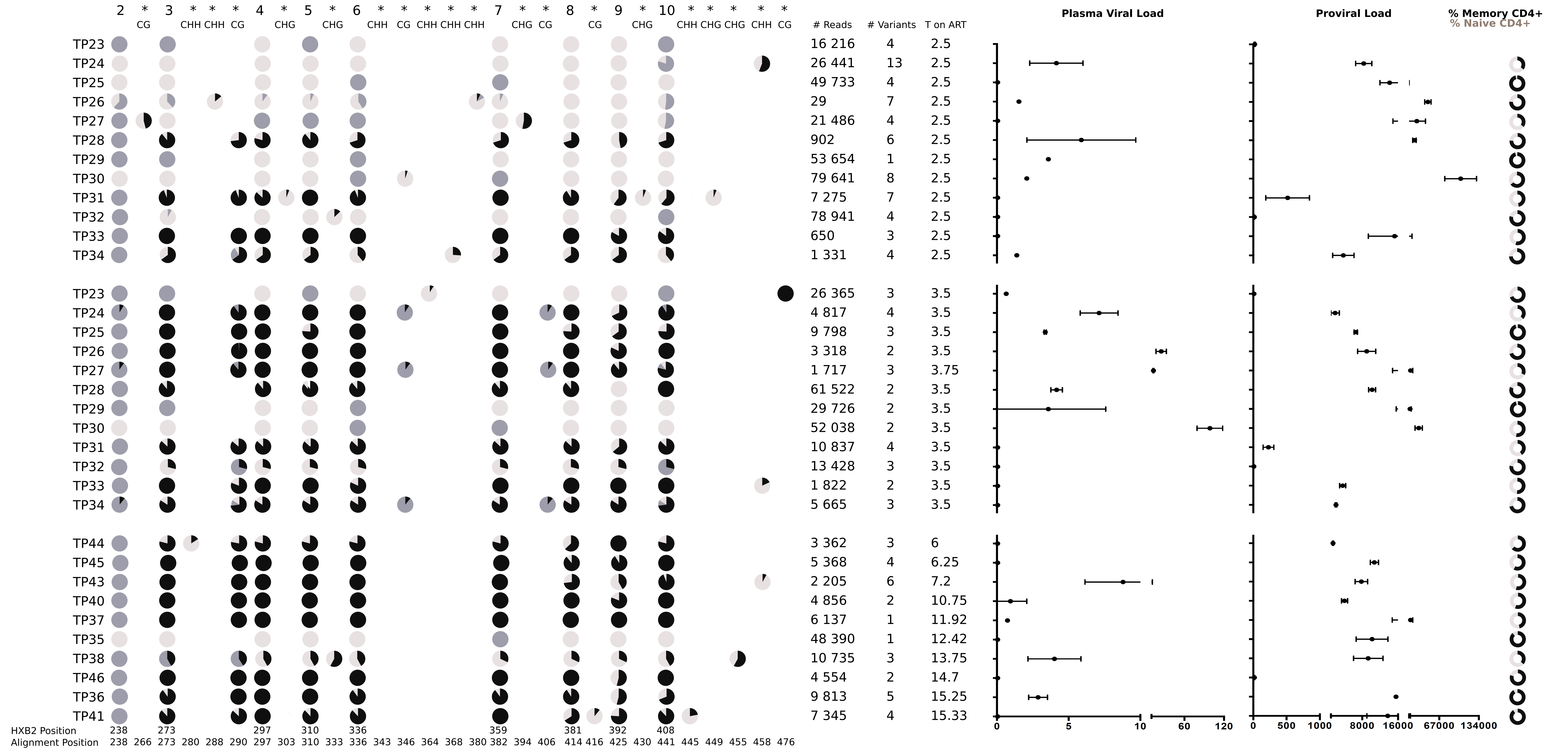

Supplement: Supplementary file 4 — Figure S3. Changes in 5’-LTR overall methylation patterns along time. A comparison of methylation patterns is shown for all participants with short-term antiretroviral therapy (ART) at 30 months (2.5 years) of follow-up (top), all participants with short-term ART at 42 months (3.5 years) of follow-up (center) and all participants with long-term ART (single time point available, 6 to 15 years) (bottom). Each line depicts a summary of all variants observed per time point. CpG sites are shown as pie charts indicating the proportion of methylated (black), unmethylated (light gray) or mutated (dark gray) variants in the sample. Canonical HXB2 CpG positions are numbered. Additional CpG, CHG and CHH methylation-susceptible sites, not observed in the HXB2 sequence, are also included (marked with *). The number of reads and the total number of variants obtained per time point, after filtering and aligning, are shown. Additionally, measurements of residual plasma viral load, proviral load and proportion of memory (CD3+/CD4+/CD28+−/CD95+; black) and naïve (CD3+/CD4+/CD45RO-/CCR7+/CD28+/CD95-; light gray) CD4+ T cells per time point are shown. (PDF 207 kb) [file 13148_2019_735_MOESM4_ESM.pdf]
