# Supplementary material for: Longitudinal variation in human immunodeficiency virus long terminal repeat methylation in individuals on suppressive antiretroviral therapy
Source: Clin Epigenetics. 2019 Sep 13;11:134. doi: 10.1186/s13148-019-0735-9 (PMC6743183; doi:10.1186/s13148-019-0735-9)
Supplement: Supplementary file 2 — Table S1. Antiretroviral treatment history of participants. (DOC 54 kb) [file 13148_2019_735_MOESM2_ESM.doc]

Table S1 – Antiretroviral treatment history of participants.

| **Patient ID** | **Initial ART** | | **ART changes** | |
| --- | --- | --- | --- | --- |
|  | **Date** | **ART regimen** | **Date** | **ART regimen** |
| **Short-Term ART Longitudinal Follow-Up Group** | | | | |
| TP23 | 02-27-12 | TDF+FTC+EFV | 08/03/12 | **ABC+3TC+EFV** |
| TP24 | 04/04/12 | TDF+FTC+EFV | 09/11/12 | **TDF+FTC+ATV/r** |
| TP25 | 04/13/12 | TDF+FTC+EFV | 12/05/12 | **ABC+3TC+EFV** |
| TP26 | 04/04/12 | **TDF+FTC+EFV** | - |  |
| TP27 | 04/26/12 | TDF+FTC+EFV | 10/29/13  08/21/14 | ABC+3TC+ATV/r  **TDF+FTC+EFV** |
| TP28 | 04/16/12 | TDF+FTC+EFV | 10/30/12  08/06/13 | TDF+FTC+ATV/r  **TDF+FTC+NVP** |
| TP29 | 04/26/12 | **TDF+FTC+EFV** | - |  |
| TP30 | 05/03/12 | **TDF+FTC+EFV** | - |  |
| TP31 | 05/14/12 | **TDF+FTC+EFV** | - |  |
| TP32 | 03/13/12 | **TDF+FTC+EFV** | - |  |
| TP33 | 05/21/12 | **TDF+FTC+EFV** | - |  |
| TP34 | 12/13/12 | **TDF+FTC+AZT+RTV** | - |  |
| **Long-Term ART Cross-Sectional Group** | | | | |
| TP35 | 03/21/04 | AZT+3TC+EFV | 08/15/13 | **TDF+FTC+EFV** |
| TP36 | 07/06/01 | AZT+3TC+IDV | 10/15/2008 | **TDF+FTC+EFV** |
| TP37 | 1997 | AZT+ddI+IDV/r | 10/15/04  11/15/04  09/15/05  03/15/09  09/15/16 | AZT+3TC+EFV  AZT+3TC+NFV  AZT+3TC+EFV+LPV/r  TDF+FTC+LPV/r  **TDF+FTC+DRV/r** |
| TP38 | 01/15/03 | AZT+3TC+EFV | 2010 | **TDF+FTC+LPV/r** |
| TP40 | 01/15/06 | AZT+3TC+EFV | 2009 | **TDF+FTC+EFV** |
| TP41 | 05/15/02 | ddI+d4T+IDV | 05/15/02  2009 | ABC+3TC+EFV  **TDF+FTC+EFV** |
| TP43 | 08/15/09 | **TDF+FTC+EFV** | - |  |
| TP44 | 11/15/10 | TDF+FTC+ATV/r | 05/15/16 | **TDF+FTC+DRV/r** |
| TP45 | 07/26/10 | **TDF+FTC+EFV** | - |  |
| TP46 | 02/19/02 | AZT+3TC+NFV | 09/15/04  10/15/09 | ddI+3TC+EFV  **TDF+FTC+EFV** |

ART, antiretroviral treatment. **Nucleoside Reverse Transcriptase Inhibitors (NRTI):** TDF, Tenofovir; FTC, Emtricitabine; AZT, Zidovudine (Azidothymidine); 3TC, Lamivudine; ddI, Didanosine; d4T, Stavudine and ABC, Abacavir. **Non-Nucleoside Reverse Transcriptase Inhibitors (NNRTI):** EFV, Efavirenz and NVP, Nevirapine. **Protease inhibitors (PI):** RTV, Ritonavir; IDV, Indinavir; NFV, Nelfinavir; ATV/r, Atazanavir/Ritonavir; DRV/r; Darunavir/Ritonavir and LPV/r, Lopinavir/Ritonavir. Current ART regimens are shown in **bold**. Participants with no ART regimen changes are shown with “-“.
