# Supplementary material for: Longitudinal variation in human immunodeficiency virus long terminal repeat methylation in individuals on suppressive antiretroviral therapy
Source: Clin Epigenetics. 2019 Sep 13;11:134. doi: 10.1186/s13148-019-0735-9 (PMC6743183; doi:10.1186/s13148-019-0735-9)
Supplement: Supplementary file 5 — Table S2. Primers for HIV 5’-LTR and methylation control amplification and sequencing. (DOCX 17 kb) [file 13148_2019_735_MOESM5_ESM.docx]

Table S2 – Primers for HIV 5’-LTR and methylation control amplification and sequencing.

| **HIV SEQUENCING PRIMERS** | | |
| --- | --- | --- |
| **Primer Name** | **Primer Sequence** | **HXB2 Positions** |
| LTR1-20Fw | TGGAAGGGCTAATTCACTCC | 1-20 |
| LTR1-20TxFw | TGGAAGGGTTAATTTATTTT | 1-20 |
| LTR835-856Rv | GCCTTAACCGAATTTTTTCCC | 835-856 |
| LTR836-856TxRv | ACCTTAACCAAATTTTTTCC | 836-856 |
| Nex-LTR213-236Fw | *TCGTCGGCAGCGTCAGATGTGTATAAGAGACAG*GTGAGCCTGCATGGAATGGATGAC | 213-236 |
| Nex-LTR213-236TxFw | *TCGTCGGCAGCGTCAGATGTGTATAAGAGACAG*GTGAGTTTGTATGGAATGGATGAT | 213-236 |
| Nex-LTR471-495Rv | *GTCTCGTGGGCTCGGAGATGTGTATAAGAGACAG*GTCTAACCAGAGAGACCCAGTACAG | 471-495 |
| Nex-LTR471-495TxRv | *GTCTCGTGGGCTCGGAGATGTGTATAAGAGACAG*ATCTAACCAAAAAAACCCAATACAA | 471-495 |
| **DAPK1 METHYLATION CONTROL PRIMERS** | | |
| **Primer Name** | **Primer Sequence** | **Gene Positions** |
| Nex-DAPK1_-867_-836Fw | *TCGTCGGCAGCGTCAGATGTGTATAAGAGACAG*ATTGGGAAGGTTAAGGYGGAGGGAAATTTGGT | -867 to -836 |
| Nex-DAPK1_-628_-594Rv | *GTCTCGTGGGCTCGGAGATGTGTATAAGAGACAG*CCCCAAACRAAACAATCCCCAAAACCACATTCCTA | -628 to -594 |

Illumina Nextera XT adapter sequences are shown in red italics. Changes in primer sequences to account for bisulfite conversion are shown in blue. Fw – Forward primers; Rv – Reverse primers; Tx – Primers for bisulfate-converted samples; LTR – long terminal repeat. The human death-associated protein kinase 1 (DAPK1) gene was used to measure bisulfate conversion efficiency. Y, R - Degenerate positions that consider methylated and unmethylated sequences.
