# Supplementary material for: Longitudinal variation in human immunodeficiency virus long terminal repeat methylation in individuals on suppressive antiretroviral therapy
Source: Clin Epigenetics. 2019 Sep 13;11:134. doi: 10.1186/s13148-019-0735-9 (PMC6743183; doi:10.1186/s13148-019-0735-9)
Supplement: Supplementary file 3 — Figure S2. Detailed HIV 5’-LTR variant composition and methylation analysis in the rest of participants. Detailed variant composition per time point is shown for ten participants with short-term antiretroviral treatment not included in Fig. 3 (TP23, TP24, TP27, TP29, TP30, TP32, TP26, TP28, TP33, TP34), and for the single time point available for the 10 participants with long-term antiretroviral therapy (TP35, TP36, TP37, TP38, TP40, TP41, TP43, TP44, TP45, TP46). Each horizontal line represents a variant. The number of reads representing that variant and its proportion in the sample are shown on the right. Each circle represents a canonical CpG site, numbered according to the HXB2 reference: black–methylated, white–unmethylated, gray–mutated. (PDF 16804 kb) [file 13148_2019_735_MOESM3_ESM.pdf]

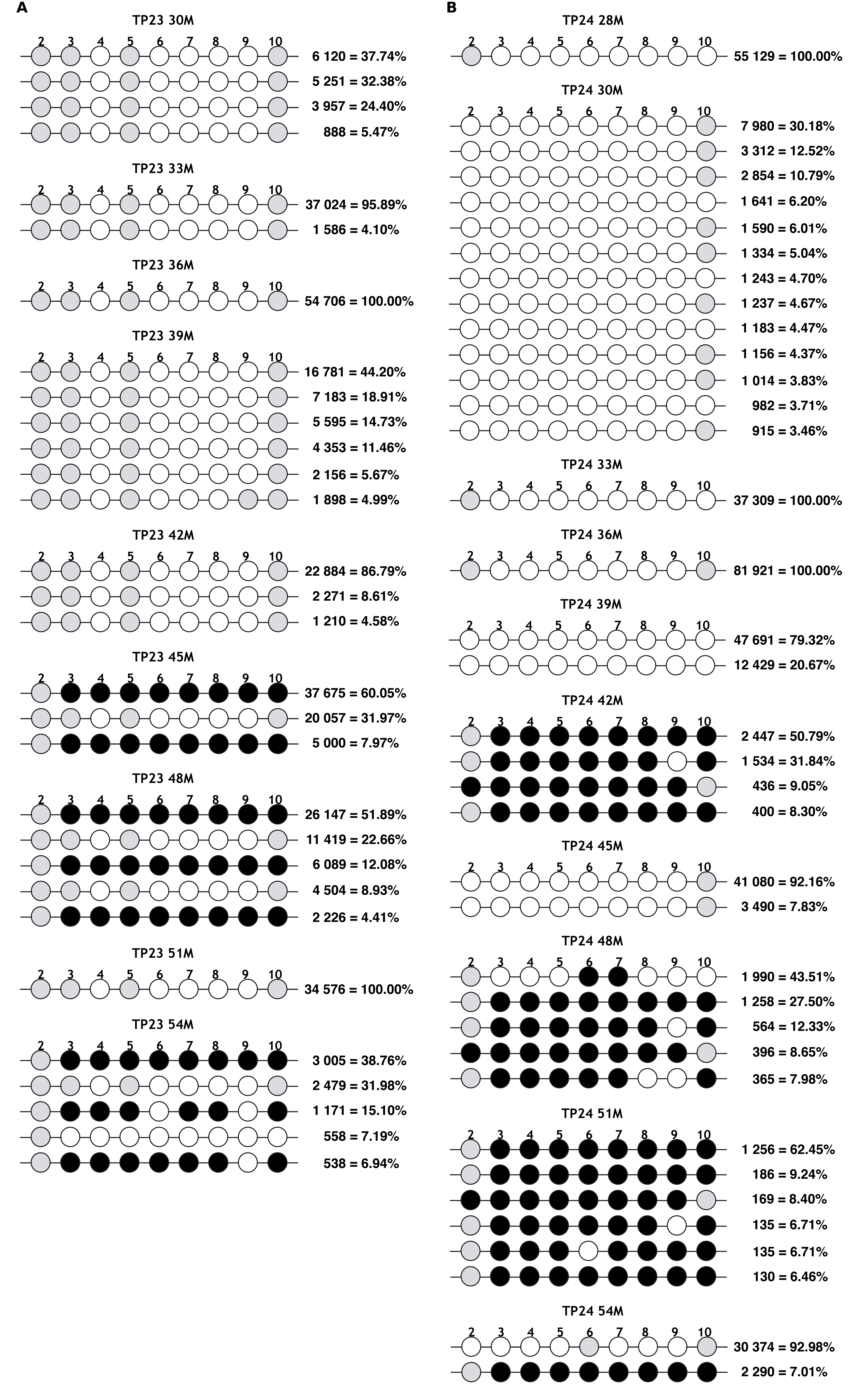
A
TP23 30M
6 120 = 37.74%
5 251 = 32.38%
3 957 = 24.40%
888 = 5.47%
TP23 33M
37 024 = 95.89%
1 586 = 4.10%
TP23 36M
54 706 = 100.00%
TP23 39M
16 781 = 44.20%
7 183 = 18.91%
5 595 = 14.73%
4 353 = 11.46%
2 156 = 5.67%
1 898 = 4.99%
TP23 42M
22 884 = 86.79%
2 271 = 8.61%
1 210 = 4.58%
TP23 45M
37 675 = 60.05%
20 057 = 31.97%
5 000 = 7.97%
TP23 48M
26 147 = 51.89%
11 419 = 22.66%
6 089 = 12.08%
4 504 = 8.93%
2 226 = 4.41%
TP23 51M
34 576 = 100.00%
TP23 54M
3 005 = 38.76%
2 479 = 31.98%
1 171 = 15.10%
558 = 7.19%
538 = 6.94%
B
TP24 28M
55 129 = 100.00%
TP24 30M
7 980 = 30.18%
3 312 = 12.52%
2 854 = 10.79%
1 641 = 6.20%
1 590 = 6.01%
1 334 = 5.04%
1 243 = 4.70%
1 237 = 4.67%
1 183 = 4.47%
1 156 = 4.37%
1 014 = 3.83%
982 = 3.71%
915 = 3.46%
TP24 33M
37 309 = 100.00%
TP24 36M
81 921 = 100.00%
TP24 39M
47 691 = 79.32%
12 429 = 20.67%
TP24 42M
2 447 = 50.79%
1 534 = 31.84%
436 = 9.05%
400 = 8.30%
TP24 45M
41 080 = 92.16%
3 490 = 7.83%
TP24 48M
1 990 = 43.51%
1 258 = 27.50%
564 = 12.33%
396 = 8.65%
365 = 7.98%
TP24 51M
1 256 = 62.45%
186 = 9.24%
169 = 8.40%
135 = 6.71%
135 = 6.71%
130 = 6.46%
TP24 54M
30 374 = 92.98%
2 290 = 7.01%

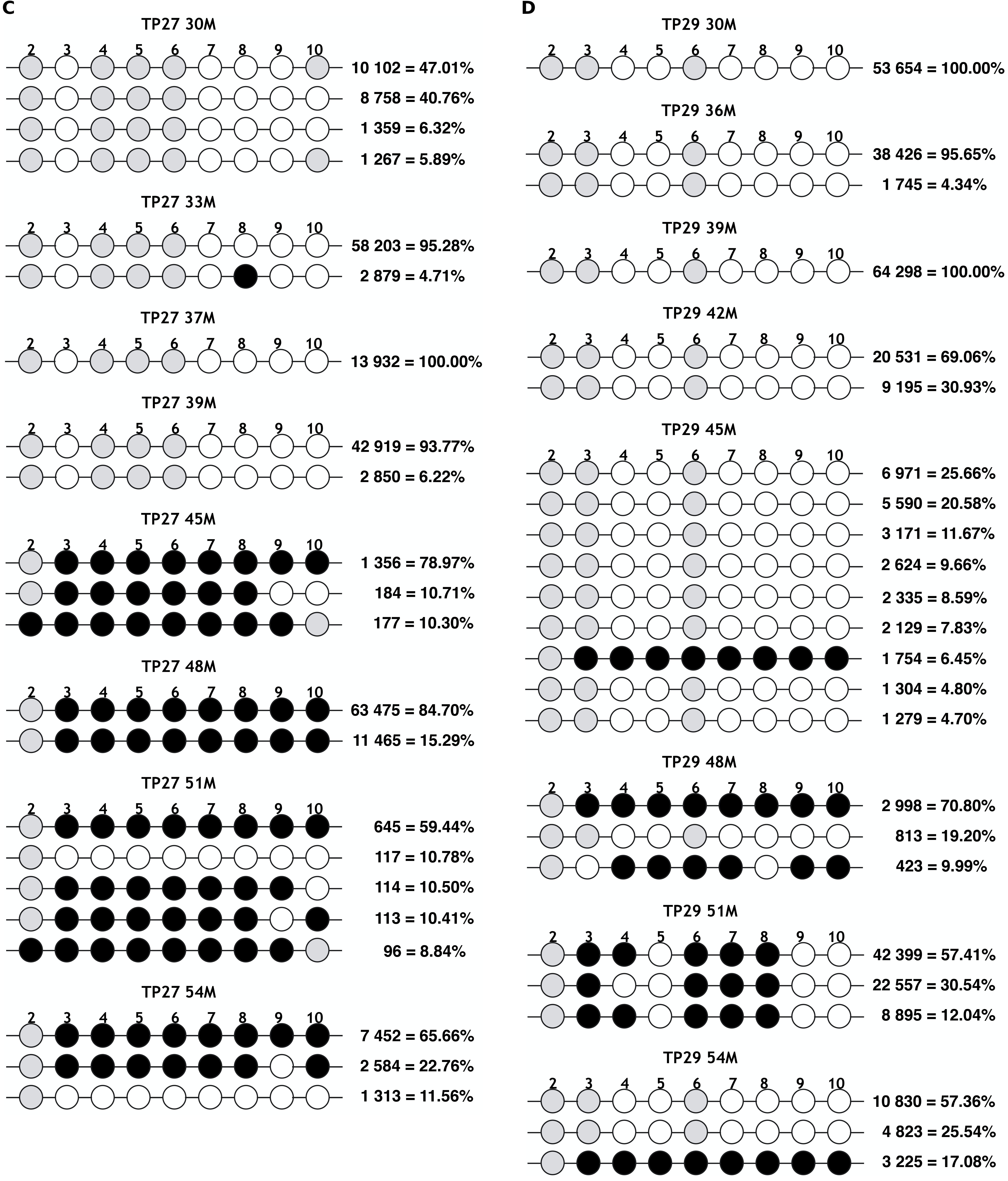
C
TP27 30M
10 102 = 47.01%
8 758 = 40.76%
1 359 = 6.32%
1 267 = 5.89%
TP27 33M
58 203 = 95.28%
2 879 = 4.71%
TP27 37M
13 932 = 100.00%
TP27 39M
42 919 = 93.77%
2 850 = 6.22%
TP27 45M
1 356 = 78.97%
184 = 10.71%
177 = 10.30%
TP27 48M
63 475 = 84.70%
11 465 = 15.29%
TP27 51M
645 = 59.44%
117 = 10.78%
114 = 10.50%
113 = 10.41%
96 = 8.84%
TP27 54M
7 452 = 65.66%
2 584 = 22.76%
1 313 = 11.56%
D
TP29 30M
53 654 = 100.00%
TP29 36M
38 426 = 95.65%
1 745 = 4.34%
TP29 39M
64 298 = 100.00%
TP29 42M
20 531 = 69.06%
9 195 = 30.93%
TP29 45M
6 971 = 25.66%
5 590 = 20.58%
3 171 = 11.67%
2 624 = 9.66%
2 335 = 8.59%
2 129 = 7.83%
1 754 = 6.45%
1 304 = 4.80%
1 279 = 4.70%
TP29 48M
2 998 = 70.80%
813 = 19.20%
423 = 9.99%
TP29 51M
42 399 = 57.41%
22 557 = 30.54%
8 895 = 12.04%
TP29 54M
10 830 = 57.36%
4 823 = 25.54%
3 225 = 17.08%

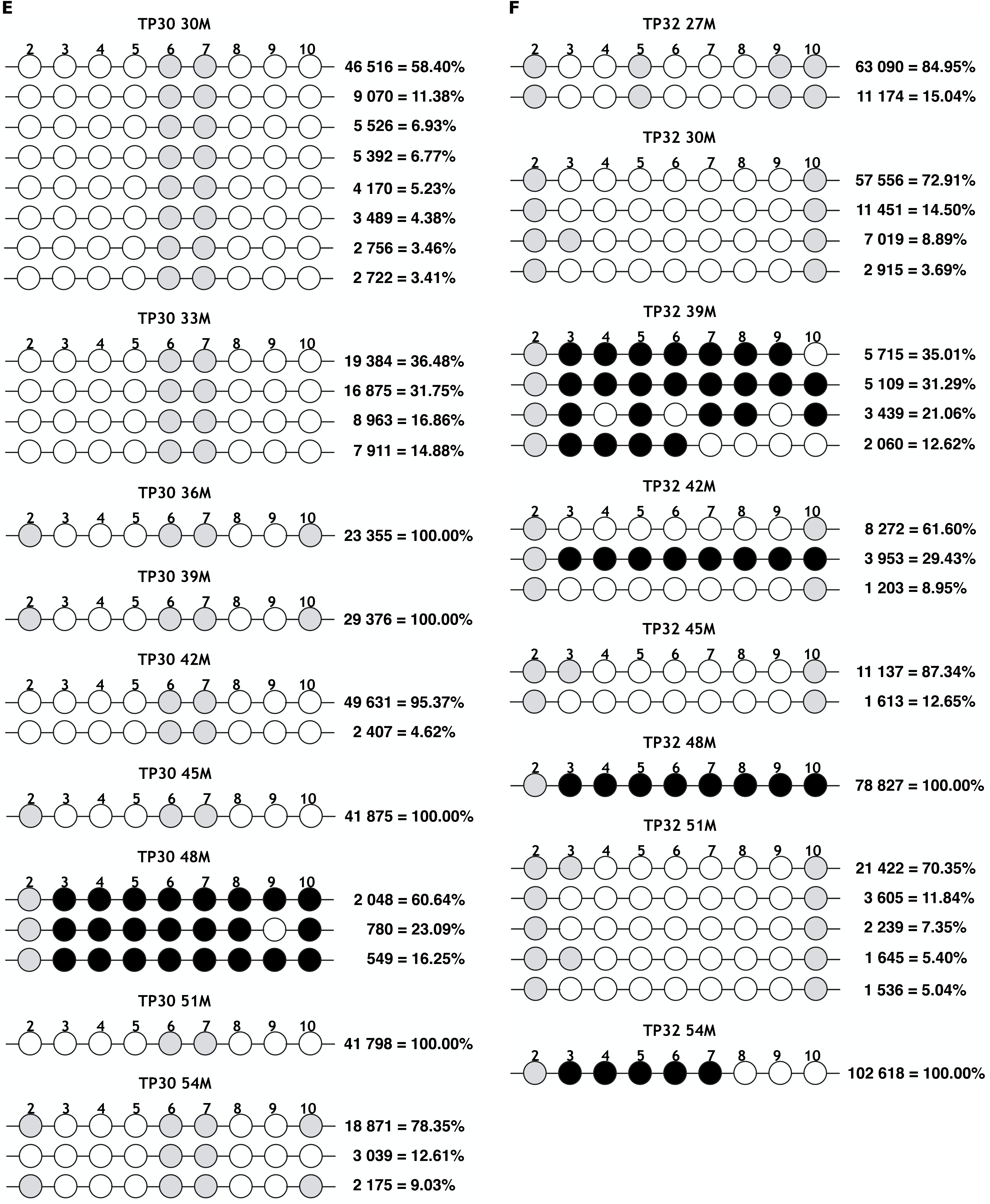
E
TP30 30M
2 3 4 5 6 7 8 9 10
46 516 = 58.40%
9 070 = 11.38%
5 526 = 6.93%
5 392 = 6.77%
4 170 = 5.23%
3 489 = 4.38%
2 756 = 3.46%
2 722 = 3.41%
TP30 33M
2 3 4 5 6 7 8 9 10
19 384 = 36.48%
16 875 = 31.75%
8 963 = 16.86%
7 911 = 14.88%
TP30 36M
2 3 4 5 6 7 8 9 10
23 355 = 100.00%
TP30 39M
2 3 4 5 6 7 8 9 10
29 376 = 100.00%
TP30 42M
2 3 4 5 6 7 8 9 10
49 631 = 95.37%
2 407 = 4.62%
TP30 45M
2 3 4 5 6 7 8 9 10
41 875 = 100.00%
TP30 48M
2 3 4 5 6 7 8 9 10
2 048 = 60.64%
780 = 23.09%
549 = 16.25%
TP30 51M
2 3 4 5 6 7 8 9 10
41 798 = 100.00%
TP30 54M
2 3 4 5 6 7 8 9 10
18 871 = 78.35%
3 039 = 12.61%
2 175 = 9.03%
F
TP32 27M
2 3 4 5 6 7 8 9 10
63 090 = 84.95%
11 174 = 15.04%
TP32 30M
2 3 4 5 6 7 8 9 10
57 556 = 72.91%
11 451 = 14.50%
7 019 = 8.89%
2 915 = 3.69%
TP32 39M
2 3 4 5 6 7 8 9 10
5 715 = 35.01%
5 109 = 31.29%
3 439 = 21.06%
2 060 = 12.62%
TP32 42M
2 3 4 5 6 7 8 9 10
8 272 = 61.60%
3 953 = 29.43%
1 203 = 8.95%
TP32 45M
2 3 4 5 6 7 8 9 10
11 137 = 87.34%
1 613 = 12.65%
TP32 48M
2 3 4 5 6 7 8 9 10
78 827 = 100.00%
TP32 51M
2 3 4 5 6 7 8 9 10
21 422 = 70.35%
3 605 = 11.84%
2 239 = 7.35%
1 645 = 5.40%
1 536 = 5.04%
TP32 54M
2 3 4 5 6 7 8 9 10
102 618 = 100.00%

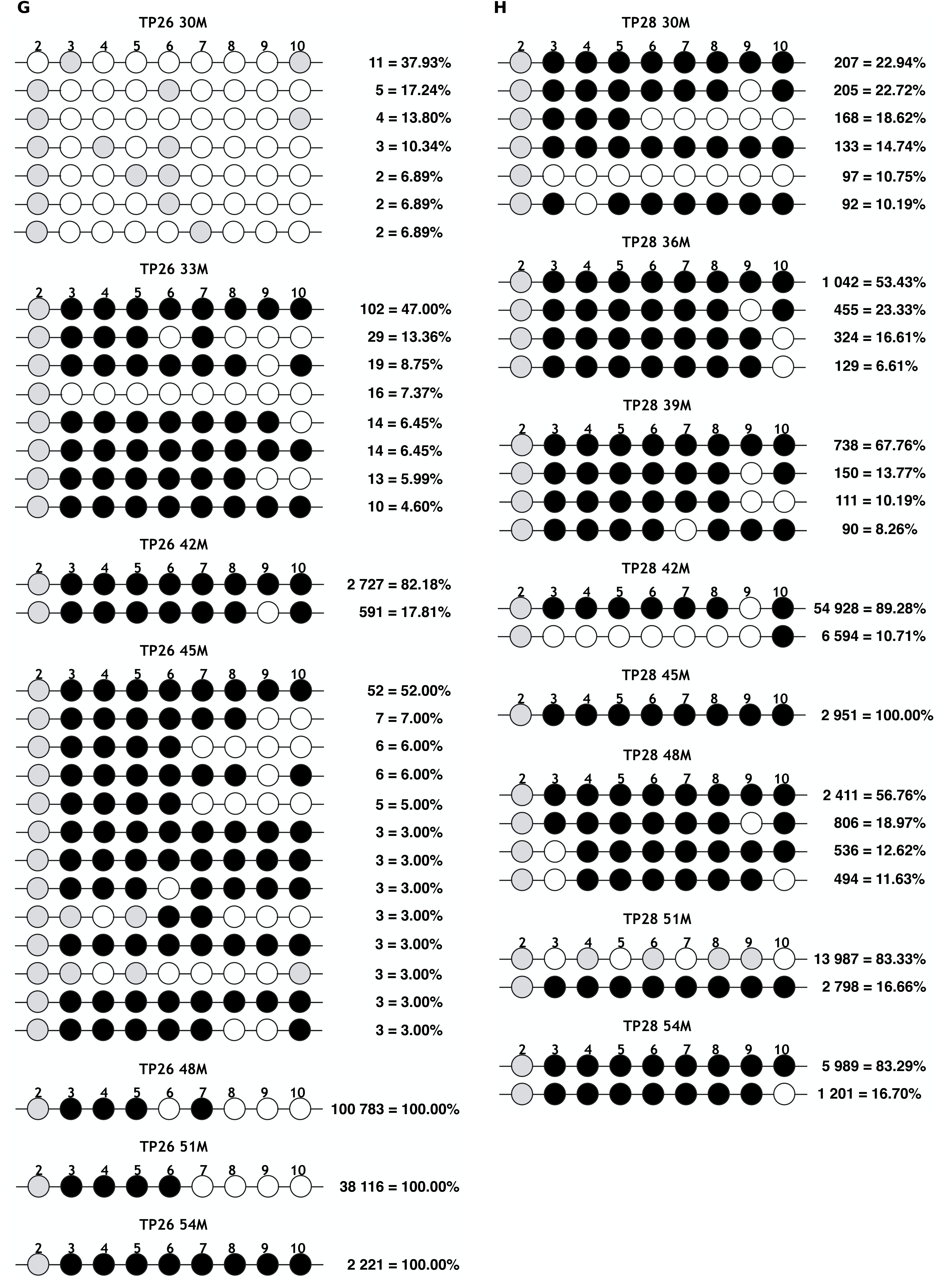
G
TP26 30M
2 3 4 5 6 7 8 9 10
11 = 37.93%
5 = 17.24%
4 = 13.80%
3 = 10.34%
2 = 6.89%
2 = 6.89%
2 = 6.89%
TP26 33M
2 3 4 5 6 7 8 9 10
102 = 47.00%
29 = 13.36%
19 = 8.75%
16 = 7.37%
14 = 6.45%
14 = 6.45%
13 = 5.99%
10 = 4.60%
TP26 42M
2 3 4 5 6 7 8 9 10
2 727 = 82.18%
591 = 17.81%
TP26 45M
2 3 4 5 6 7 8 9 10
52 = 52.00%
7 = 7.00%
6 = 6.00%
6 = 6.00%
5 = 5.00%
3 = 3.00%
3 = 3.00%
3 = 3.00%
3 = 3.00%
3 = 3.00%
3 = 3.00%
3 = 3.00%
3 = 3.00%
TP26 48M
2 3 4 5 6 7 8 9 10
100 783 = 100.00%
TP26 51M
2 3 4 5 6 7 8 9 10
38 116 = 100.00%
TP26 54M
2 3 4 5 6 7 8 9 10
2 221 = 100.00%
H
TP28 30M
2 3 4 5 6 7 8 9 10
207 = 22.94%
205 = 22.72%
168 = 18.62%
133 = 14.74%
97 = 10.75%
92 = 10.19%
TP28 36M
2 3 4 5 6 7 8 9 10
1 042 = 53.43%
455 = 23.33%
324 = 16.61%
129 = 6.61%
TP28 39M
2 3 4 5 6 7 8 9 10
738 = 67.76%
150 = 13.77%
111 = 10.19%
90 = 8.26%
TP28 42M
2 3 4 5 6 7 8 9 10
54 928 = 89.28%
6 594 = 10.71%
TP28 45M
2 3 4 5 6 7 8 9 10
2 951 = 100.00%
TP28 48M
2 3 4 5 6 7 8 9 10
2 411 = 56.76%
806 = 18.97%
536 = 12.62%
494 = 11.63%
TP28 51M
2 3 4 5 6 7 8 9 10
13 987 = 83.33%
2 798 = 16.66%
TP28 54M
2 3 4 5 6 7 8 9 10
5 989 = 83.29%
1 201 = 16.70%

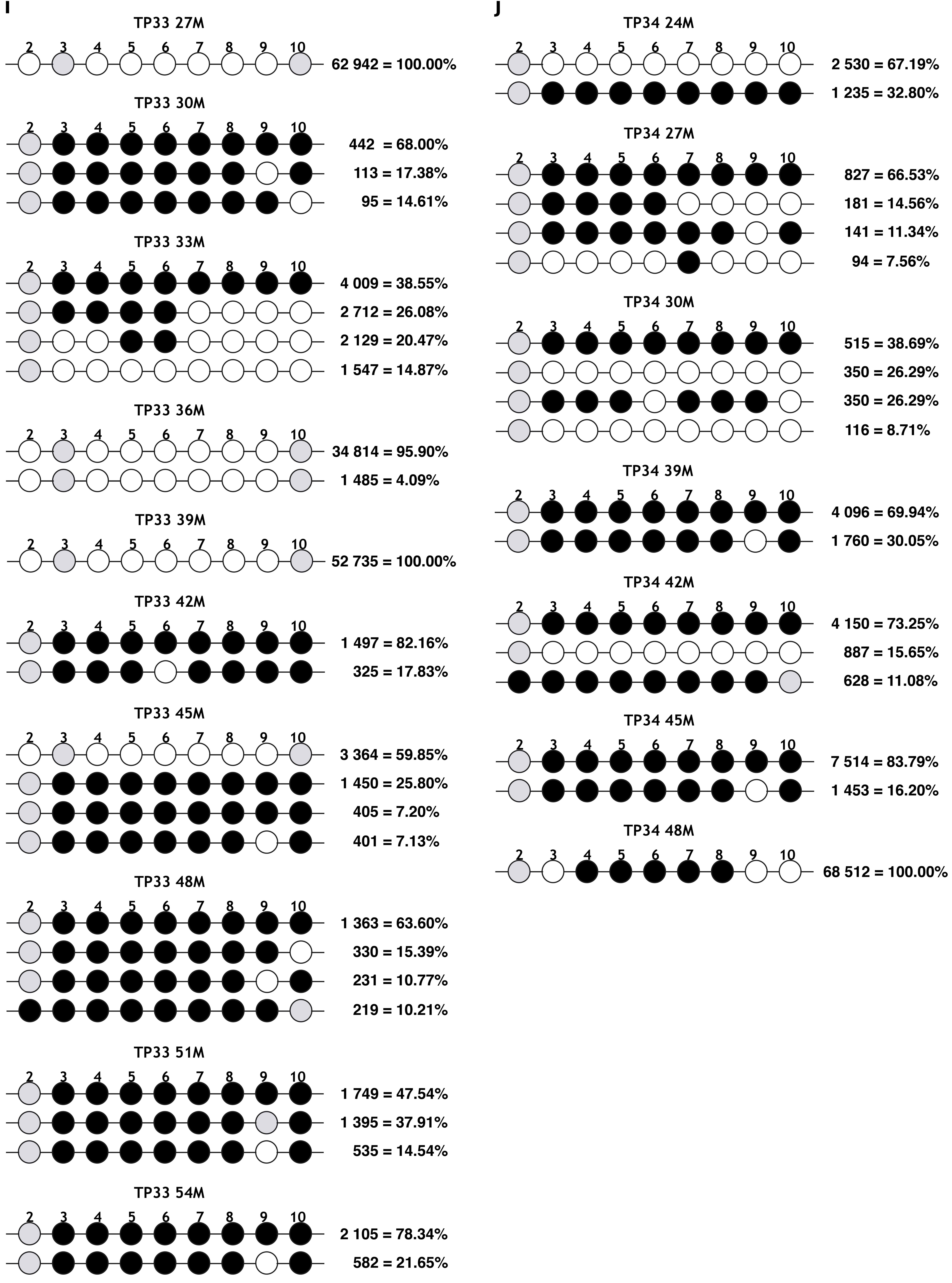
I
TP33 27M
2 3 4 5 6 7 8 9 10
62 942 = 100.00%
TP33 30M
2 3 4 5 6 7 8 9 10
442 = 68.00%
113 = 17.38%
95 = 14.61%
TP33 33M
2 3 4 5 6 7 8 9 10
4 009 = 38.55%
2 712 = 26.08%
2 129 = 20.47%
1 547 = 14.87%
TP33 36M
2 3 4 5 6 7 8 9 10
34 814 = 95.90%
1 485 = 4.09%
TP33 39M
2 3 4 5 6 7 8 9 10
52 735 = 100.00%
TP33 42M
2 3 4 5 6 7 8 9 10
1 497 = 82.16%
325 = 17.83%
TP33 45M
2 3 4 5 6 7 8 9 10
3 364 = 59.85%
1 450 = 25.80%
405 = 7.20%
401 = 7.13%
TP33 48M
2 3 4 5 6 7 8 9 10
1 363 = 63.60%
330 = 15.39%
231 = 10.77%
219 = 10.21%
TP33 51M
2 3 4 5 6 7 8 9 10
1 749 = 47.54%
1 395 = 37.91%
535 = 14.54%
TP33 54M
2 3 4 5 6 7 8 9 10
2 105 = 78.34%
582 = 21.65%
J
TP34 24M
2 3 4 5 6 7 8 9 10
2 530 = 67.19%
1 235 = 32.80%
TP34 27M
2 3 4 5 6 7 8 9 10
827 = 66.53%
181 = 14.56%
141 = 11.34%
94 = 7.56%
TP34 30M
2 3 4 5 6 7 8 9 10
515 = 38.69%
350 = 26.29%
350 = 26.29%
116 = 8.71%
TP34 39M
2 3 4 5 6 7 8 9 10
4 096 = 69.94%
1 760 = 30.05%
TP34 42M
2 3 4 5 6 7 8 9 10
4 150 = 73.25%
887 = 15.65%
628 = 11.08%
TP34 45M
2 3 4 5 6 7 8 9 10
7 514 = 83.79%
1 453 = 16.20%
TP34 48M
2 3 4 5 6 7 8 9 10
68 512 = 100.00%

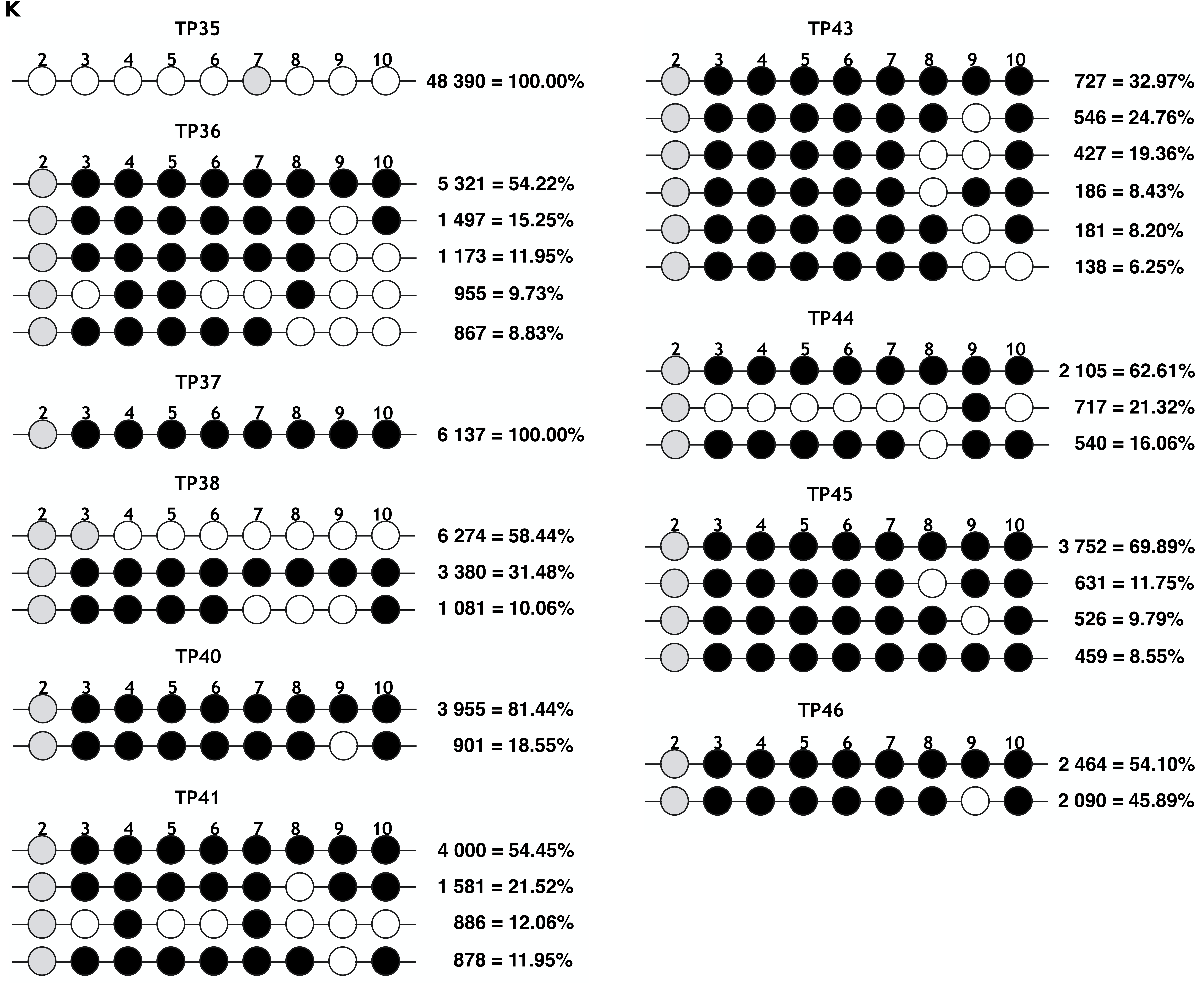
K
TP35
48 390 = 100.00%
TP36
5 321 = 54.22%
1 497 = 15.25%
1 173 = 11.95%
955 = 9.73%
867 = 8.83%
TP37
6 137 = 100.00%
TP38
6 274 = 58.44%
3 380 = 31.48%
1 081 = 10.06%
TP40
3 955 = 81.44%
901 = 18.55%
TP41
4 000 = 54.45%
1 581 = 21.52%
886 = 12.06%
878 = 11.95%
TP43
727 = 32.97%
546 = 24.76%
427 = 19.36%
186 = 8.43%
181 = 8.20%
138 = 6.25%
TP44
2 105 = 62.61%
717 = 21.32%
540 = 16.06%
TP45
3 752 = 69.89%
631 = 11.75%
526 = 9.79%
459 = 8.55%
TP46
2 464 = 54.10%
2 090 = 45.89%
